# Long non-coding RNAs in ferroptosis and cuproptosis impact on prognosis and treatment in hepatocellular carcinoma

**DOI:** 10.1007/s10238-024-01397-x

**Published:** 2024-06-22

**Authors:** Kun Wang, Chunqian Yang, Jingen Xie, Xiao Zhang, Ting Wei, Zhu Yan

**Affiliations:** 1 Department of Gastroenterology, The First People’s Hospital of Lianyungang, Lianyungang, China; 2 Department of Oncology, Zhujiang Hospital, Southern Medical University, Guangzhou, China; 3 Department of General Medicine, Huai’an Cancer Hospital, Huai’an, China; 4 Department of Thoracic Surgery, The First Affiliated Hospital of Nanjing Medical University, Nanjing, China; 5 Emergency Medicine Department, Huai’an Hospital Affiliated to Yangzhou University (The Fifth People’s Hospital of Huai’an), Huaian, China

**Keywords:** HCC, Cuproptosis, Ferroptosis, lncRNA, qRT-PCR, Elesclomol, Erastin

## Abstract

Ferroptosis and cuproptosis are recently discovered forms of cell death that have gained interest as potential cancer treatments, particularly for hepatocellular carcinoma. Long non-coding RNAs (lncRNAs) influence cancer cell activity by interacting with various nucleic acids and proteins. However, the role of ferroptosis and cuproptosis-related lncRNAs (FCRLs) in cancer remains underexplored. Ferroptosis and cuproptosis scores for each sample were assessed using Gene Set Variation Analysis (GSVA). Weighted correlation network analysis identified the FCRLs most relevant to our study. A risk model based on FCRLs was developed to categorize patients into high-risk and low-risk groups. We then compared overall survival (OS), tumor immune microenvironment, and clinical characteristics between these groups. The IPS score and ImmuCellAI webpage were used to predict the association between FCRL-related signatures and immunotherapy response. Finally, we validated the accuracy of FCRLs in hepatocellular carcinoma cell lines using induction agents (elesclomol and erastin). Patients in different risk subgroups showed significant differences in OS, immune cell infiltration, pathway activity, and clinical characteristics. Cellular assays revealed significant changes in the expression of AC019080.5, AC145207.5, MIR210HG, and LINC01063 in HCC cell lines following the addition of ferroptosis and cuproptosis inducers. We created a signature of four FCRLs that accurately predicted survival in HCC patients, laid the foundation for basic research related to ferroptosis and cuproptosis in hepatocellular carcinoma, and provided therapeutic recommendations for HCC patients.

**Supplementary Information:**

The online version contains supplementary material available at 10.1007/s10238-024-01397-x.

## Introduction

Liver cancer is the most widespread type of cancer globally and a leading cause of cancer deaths in China [1, 2]. The majority of liver cancer cases, 75–85%, are classified as hepatocellular carcinoma (HCC) [3]. Unfortunately, HCC often goes undetected until it has advanced, as its symptoms are not immediately noticeable, leading to a low cure rate [4]. Despite advances in HCC treatment, such as the use of PD-1/PD-L1 inhibitors, patients with advanced HCC continue to face poor outcomes due to its metastatic and recurring nature [5]. The diverse nature of HCC across individuals and different sites highlights the urgency for identifying new, dependable biomarkers.

Ferroptosis, a type of programmed cell death driven by iron-dependent lipid peroxidation, heavily influences tumor biology and treatment [6]. There is an increasing amount of mechanistic research in gastrointestinal tumors currently [7–12]. Iron metabolism disorders have a close link to cancer, leading to an extensive study on iron-dependent toxicity [13, 14]. Copper ions also play a role in cell death similar to iron ions. High intracellular copper levels result in the same cytotoxicity as seen in ferroptosis, known as cuproptosis. This death mechanism is controlled by copper ions and caused by their direct attachment to the tricarboxylic acid cycle pathway, leading to protein aggregation and proteotoxic stress response [15]. Recent studies have found that copper ions play a role in cell death similar to that of iron ions. When intracellular concentrations of copper ions exceed the threshold for maintaining homeostatic mechanisms, the same cytotoxicity is exhibited. It was discovered that the unusual cell death mechanism known as cuproptosis depends on and is controlled by the copper ions in the cell [16, 17]. When copper ions bind to lipid-acylated elements present in the tricarboxylic acid cycle pathway, it can trigger the accumulation of lipid-acylated proteins while simultaneously reducing the levels of iron–sulfur cluster proteins. This can ultimately lead to the activation of a proteotoxic stress response within the cell, which can result in cell death. It is crucial to understand the molecular mechanisms underlying these processes, as they can have significant implications for human health and disease. This process is known as cuproptosis, and it has been implicated in the pathogenesis of various diseases, including cancer. Moreover, recent research has shown that risk signatures associated with ferroptosis and cuproptosis are not only associated with prognosis and immunity in HCC but also suggest that the four risk genes comprising the signature may play a role in shaping the tumor microenvironment (TME) of HCC [17, 18]. Studies have shown that cancer patients have significantly higher levels of copper in both their blood and tumor tissue compared to healthy individuals [19]. Despite this, there is still ongoing research into the use of copper ion carriers and chelators in anti-cancer therapy [20, 21]. The workings behind cuproptosis in malignancies, as well as its potential connection to ferroptosis, remain subjects of the ongoing investigation.

Long non-coding RNAs (lncRNAs) are a type of non-coding RNA that is longer than 200 nt and is an essential component of the non-coding genome [22]. Most lncRNAs are specifically expressed in tumors and regulate a variety of tumor biological processes [22]. Some lncRNAs have been identified in recent investigations. Recent research has shown certain lncRNAs as novel targets for the development of HCC, which will give new targets for the therapy of HCC on an individual basis [23].

Research on lncRNAs associated with ferroptosis (FRL) and cuproptosis (CRL) has been extensively conducted [24]. However, whether lncRNAs simultaneously regulate both ferroptosis and cuproptosis remains unclear. The impact of ferroptosis and cuproptosis-related lncRNAs (FCRLs) on HCC prognosis and their role in modulating the TME are yet to be determined. To enhance prognosis prediction and the effectiveness of immunotherapy in HCC patients, we developed a unique FCRL prediction model using Gene Set Variation Analysis (GSVA) and Weighted Gene Co-Expression Network Analysis (WGCNA). In the TCGA-LIHC cohort, we identified four FCRLs and explored their relationships with immunotherapy, TME immune characteristics (TIME), and chemotherapy sensitivity. Additionally, we investigated the functions of these four FCRLs in regulating ferroptosis and cuproptosis through quantitative real-time polymerase chain reaction (qRT-PCR) in hepatocellular carcinoma cell lines and normal hepatocytes. Our aim is to advance the understanding of HCC's lncRNA expression profile and identify potential biomarkers for informed treatment planning.

## Materials and methods

### Raw data acquisition

**Fig. 1:**
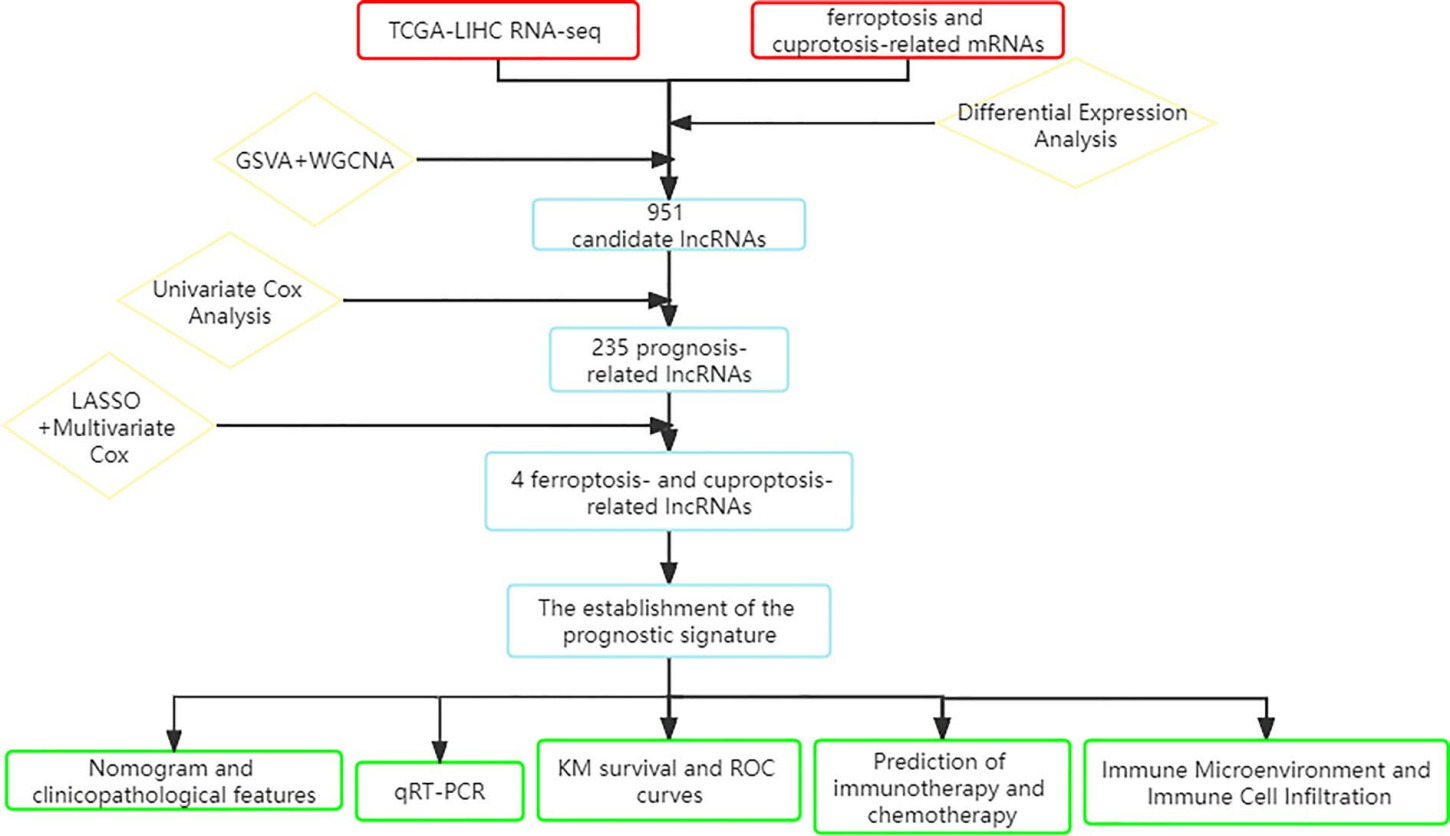
The flowchart

We downloaded transcriptional profiles, mutational data, and clinicopathological characteristics of the TCGA-LIHC cohort from the UCSC Xena website (https://xena.ucsc.edu/). Fifty samples of healthy liver tissue and 374 samples of liver cancer tissue were included in the TCGA cohort. From previous studies, we obtained 19 cuproptosis-related genes (CRG). From the FerrDb portal (http://www.zhounan.org/ferrdb) [25], we obtained 369 genes that induce ferroptosis, 349 genes that suppress ferroptosis, and 11 marker genes for ferroptosis, collectively known as ferroptosis-related genes (FRG). Finally, we calculated the characteristic scores of these two significant gene sets in the TCGA cohort using the "GSVA" approach [26, 27]. The flowchart of this work is shown in Fig. 1.

Recognition of important co-expression modules.

WGCNA [28] is a systems biology method designed to discern complex patterns of gene relationships across various samples. This analytical approach involves constructing a network of genes based on the strength of their correlations. Unlike simple clustering, WGCNA constructs a weighted network where connections between genes are represented as edges with weights proportional to the correlation between gene expressions. The process begins by calculating the pairwise correlation between all gene pairs, from which an adjacency matrix is derived. This matrix is then transformed into a topological overlap matrix that measures not just the direct interactions between genes but also the extent to the shared neighbors, enhancing the network's robustness by highlighting highly interconnected gene modules. These modules are detected through hierarchical clustering, based on the topological overlap, followed by dynamic tree cutting to define module boundaries. Each module is then related to external clinical traits (phenotypes), allowing researchers to identify modules whose expression patterns are closely associated with specific phenotypes.

### Enrichment analysis and differential lncRNAs

With |log2FC| > 1.0 and FDR < 0.05 as cut-off values, differential expression analysis was carried out to filter differentially expressed lncRNAs in the TCGA cohort [29]. Using GSEA software 4.1.0 [30], we performed Gene Set Enrichment Analyses (GSEA) and discovered significantly enriched pathways between the LR and HR groups.

### Development of FCRL-related prognostic models

We divided patients in the TCGA-LIHC cohort randomly into two groups, a training group (*n* = 185) and a test group (*n* = 185), using a 1:1 ratio with the help of the "caret" R package. Univariate Cox analysis was conducted to identify prognosis-related genes from the lncRNAs in the training group. The number of lncRNAs was then reduced with the assistance of the "glmnet" R package, and the Least Absolute Shrinkage and Selection Operator (LASSO) regression analysis was used to select the appropriate variables [31, 32]. Multivariate Cox regression analysis was then performed on the lncRNAs selected by LASSO regression, which resulted in the calculation of a risk score by summing the expression of each gene multiplied by its corresponding risk factor (risk score = Σ(Expi * coefi)). All patients were categorized as either high-risk (HR) or low-risk (LR) groups based on the median risk score of the training group. To visualize the differences in survival and patient status, we used the "survminer" and "ggrisk" R packages to create survival curves and risk maps, respectively. To evaluate the accuracy of the risk ratings in predicting one-year, three-year, and five-year OS in LIHC patients, we employed the "timeROC" package of R software to generate subject operating characteristic curves.

### Estimation of the tumor immune microenvironment.

The immune score and matrix score of each LIHC sample were assessed using the R program "ESTIMATE" [33] to quantify the number of immune and matrix components present in vivo. ssGSEA [26] is a GSEA analysis performed on a single sample, and how the gene list is ordered and the ES is calculated is dependent on the expression values of the genes in the sample, and no longer on the correlation of the genes with the phenotype. We used currently used algorithms for immune cell infiltration, including XCELL [34], TIMER [35], QUANTISEQ, MCPCOUNT, EPIC [36], CIBERSORT [37], and CIBERSORT-ABS.

### Immunotherapeutic response prediction and drug sensitivity.

The Cancer Immunome Atlas (TCIA) online software presents extensive immunogenomic analysis results. The Immunophenotype Score (IPS) evaluates the immunogenicity of tumors using a scale of 0–10 [38]. We gathered relevant data on immunotherapy in patients with LIHC using the ImmuCellAI portal (http://bioinfo.life.hust.edu.cn/ImmuCellAI), which is a tool for estimating tumor immune infiltration estimates and immunotherapy response [39]. The "pRRophetic" R [40, 41] software primarily functions to predict phenotypes from gene expression data and drug sensitivity in external cell lines using information from the Cancer Genome Project CGP cell lines (CCLE) [42].

### Construction of ferroptosis and cuproptosis model.

Normal hepatocytes LO2 and human hepatocellular carcinoma cell line HuH7 were purchased from the cell bank of Shanghai Cell Institute (Shanghai, China). All cells were grown in a defined DMEM medium (Sangon Biotech, China) containing 10% fetal bovine serum (FBS, Sangon Biotech, China) at 37 °C and 5% CO_2_. Consistent with the published literature [43], a 2h pulse treatment with elesclomol-2-CuCl2 (100 nM elesclomol + 1 m CuCl_2_) induced the onset of cuproptosis in HuH7 cells. Erastin can trigger the onset of oxidative and iron-dependent cell death [44]. With RSL molecule, erastin (10 μM) treatment of HuH7 cells resulted in a time-dependent increase in cytosolic and lipid ROS starting at 2 h. After 24 h, the total RNA was extracted with a TRIzol reagent. First, total RNA was extracted by qRT-PCR to determine the difference between LO2 and HuH7 related to cuproptosis and ferroptosis and the differences in the expression of lncRNAs. Changes in the expression of lncRNAs associated with cuproptosis and ferroptosis in HuH7 cells before and after drug treatment were determined by qRT-PCR. qRT-PCR test primer sequences are supplemented in Supplementary Table 1. Finally, they were plotted by GraphPad Prism software.

### Statistical analysis

R version 4.1.0, 64-bit6, and its support package were used for all analyses. To assess differences between subgroups, Wilcoxon tests were employed, and p values were adjusted using the BH technique. The "prcomp" function and the "stats" package were used to perform principal component analysis (PCA) [45]. Analysis by Spearman was utilized to evaluate correlation coefficients. In each statistical analysis, a *p* value of 0.05 was regarded as statistically significant.

## Results

### Identification of candidate FCRLs

**Fig. 2:**
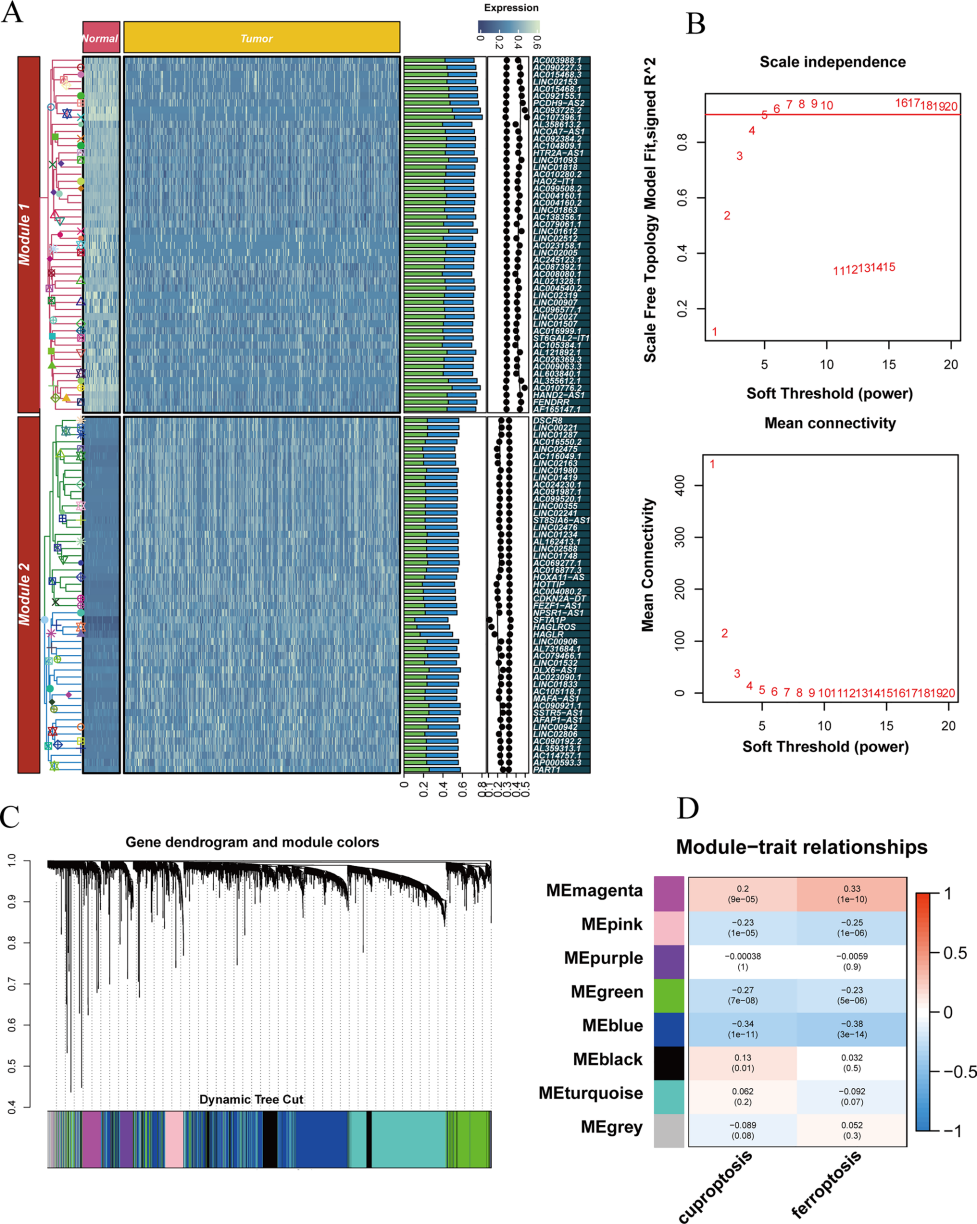
Identification of candidate ferroptosis and cuproptosis-related lncRNAs. **A** Heat map of differentially expressed lncRNAs in the TCGA cohort. **B** Scale independence and average connectivity. **C** Cluster dendrogram. **D** Heat map of correlation between modules and two apoptotic modalities

We isolated mRNA and lncRNA expression profiles from the TCGA-LIHC cohort, resulting in 13,481 lncRNAs. Differential expression analysis identified 3,438 DEGs, of which 96 were down-regulated in tumor tissues, while the remaining 3,342 were up-regulated in liver cancer tissues. The heat map in Fig. 2A displays the top 50 up-regulated and 50 down-regulated genes with the largest expression differences. Using GSVA, we calculated ferroptosis and cuproptosis-related scores for each sample and identified key modules associated with these processes via co-expression network analysis. The soft threshold power *β* was 5 when the scale-free topology fit index reached 0.9 during network construction (Fig. 2B), resulting in eight modules using the "merged dynamic" algorithm (Fig. 2C). Two modules (magenta and blue) showed the strongest correlation with ferroptosis and cuproptosis-related scores (Fig. 2D, *p* < 0.001) and were selected as key modules. We obtained 951 candidate lncRNAs by taking the intersection of genes in these modules.

### The establishment of FCRL-related prognostic signatures in LIHC

**Fig. 3:**
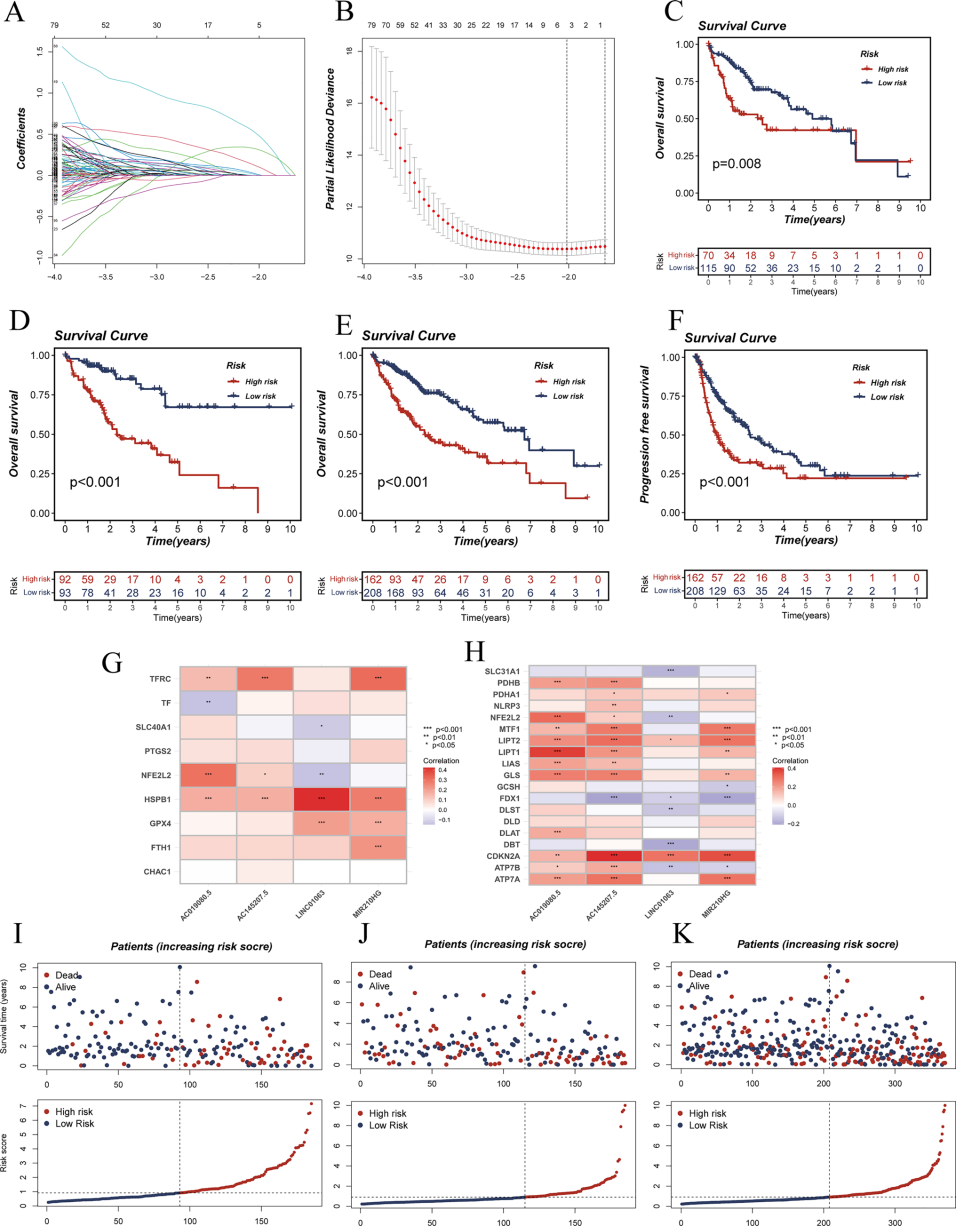
Construction and development of a prognostic model for patients with LIHC. **A** and **B** Six FCRLs were selected for multivariate analysis based on the results of the LASSO regression analysis. **C**, **D**, and **E** Survival curves to assess the risk stratification ability of the training set, validation set, and the whole TCGA cohort. **F** Progression-free survival curves for the whole cohort. **G** Correlation of four FCRLs and nine marker genes for ferroptosis. **H** Correlation of four FCRLs and 19 cuproptosis-associated genes. **I**, **J**, and **K** Risk plots are used to illustrate the survival status of each sample in the training set, validation set, and the entire TCGA cohort

Initially, the TCGA cohort of patients was divided into a training group (*n* = 185) and a test group (*n* = 185) randomly using the "caret package" in a 1:1 ratio (Supplementary Table 2). The training group was then subjected to univariate Cox regression analysis, identifying 235 lncRNAs related to OS (Supplementary Table 3). Moreover, LASSO regression revealed six lncRNAs significantly linked to ferroptosis and cuproptosis, both associated with prognosis in LIHC patients (Fig. 3A and B). Of these, multivariate Cox analysis determined that four lncRNAs, namely AC019080.5, AC145207.5, MIR210HG, and LINC01063, were associated with prognosis. A risk score was computed using the following formula: risk score = (expression level of AC019080.50.54) + (expression level of AC145207.50.58) + (expression level of MIR210HG0.23) + (expression level of LINC010630.37).

In this study, the patients in both the training and validation sets were divided into HR and LR groups based on the median value of the risk scores. The HR group of the training set had shorter overall survival durations than the LR group (*p* = 0.008, Fig. 3C). This finding was similarly verified in the validation set (*p* < 0.001, Fig. 3D) and the overall TCGA cohort (*p* < 0.001, Fig. 3E). Moreover, patients with hepatocellular carcinoma in the HR group had worse progression-free survival (PFS) than those in the LR group (*p* < 0.001, Fig. 3F). The distribution of risk scores for survival time and survival status between the HR and LR groups in the training set demonstrated that patients in the HR group had worse prognostic outcomes (Fig. 3I), and this result was further validated in the validation set and the entire TCGA cohort (Fig. 3J and K). We assessed the correlation of nine marker genes regarding ferroptosis and 19 genes regarding cuproptosis with the four models on lncRNA expression, respectively. The lncRNAs involved in the construction of the signature are likely to be involved in the biological progression of both ferroptosis and cuproptosis in LIHC, as shown in Fig. 3G and H. 

### Prognostic signature associated with FCRL is an independent predictor

**Fig. 4:**
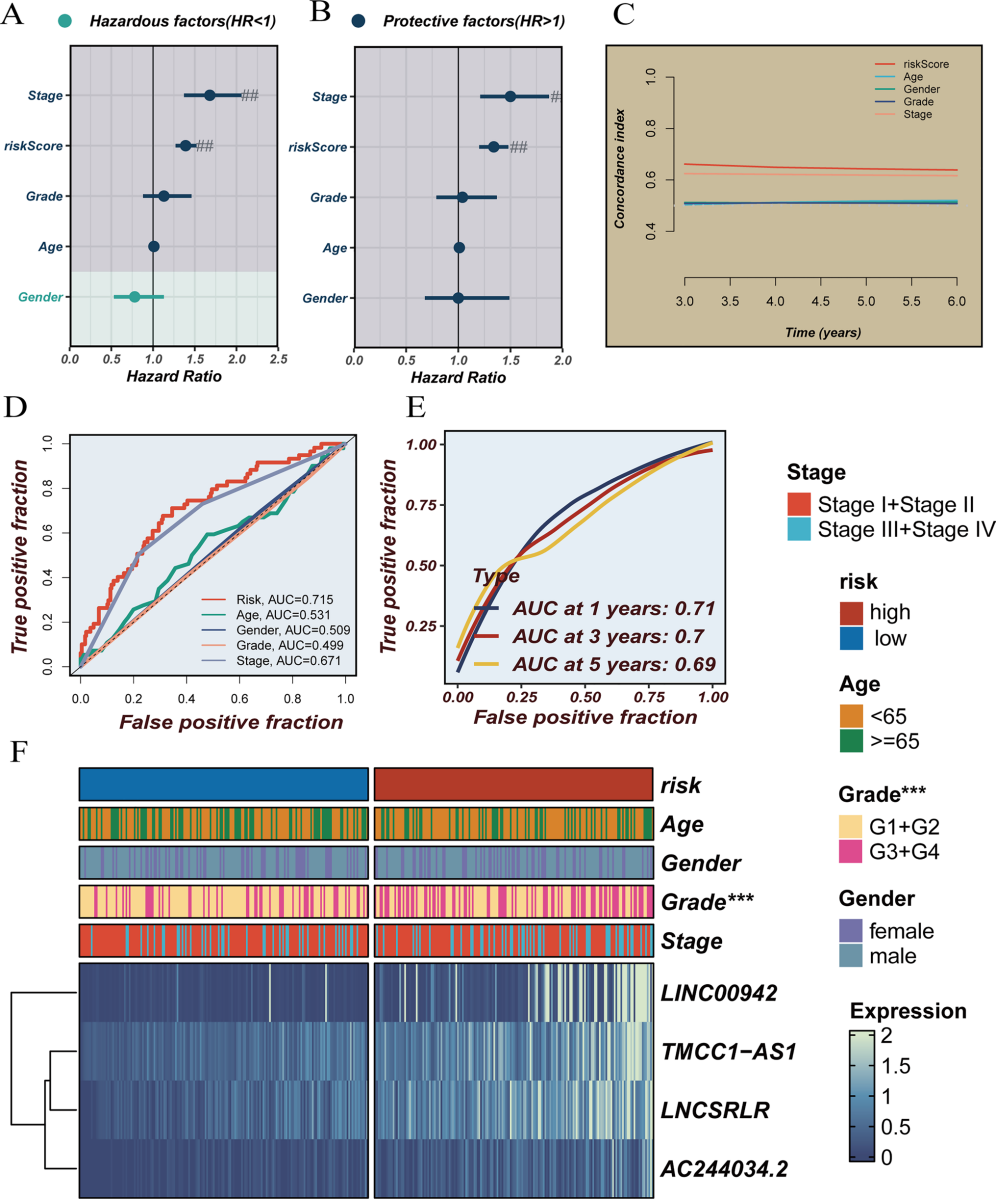
Prognostic value of risk scores in patients with LIHC. **A** Univariate and **B** multivariate Cox analysis to assess prognostic characteristics and clinical features (including age, sex, grade, and stage). **C** The risk score and C-index of clinical characteristics. **D** AUC values for risk score and clinical characteristics. **E** ROC curves for risk scores at one, three, and five years. **F** Heat map describing the relationship between risk scores and clinicopathological characteristics with different expressions of the four FCRLs between different risk subgroups. Note: # indicates *p* < 0.05; ## indicates *p* < 0.01

Univariate Cox regression analysis revealed that among all clinical factors, only tumor stage and the risk score we created for LIHC patients were linked to OS (Fig. 4A). It was further discovered through multivariate Cox regression analysis that tumor stage and risk score continued to serve as prognostic markers for LIHC patients (Fig. 4B). The FCRL risk score model outperformed the standard clinicopathological characteristics in predicting the prognosis of LIHC, as demonstrated by the C-index and the one-year ROC curve (AUC = 0.715) (Fig. 4C and D). The FCRL risk score's one-year, three-year, and five-year OS prediction AUC values for the full TCGA cohort were, respectively, 0.715, 0.698, and 0.651, which shows the prediction model has excellent specificity and sensitivity (Fig. 4E). The heat map in Fig. 4F shows the gender, age, grading, staging, and risk scores of the patient sample for all TCGA cohorts.

### Correlation analysis of clinicopathological features

**Fig. 5:**
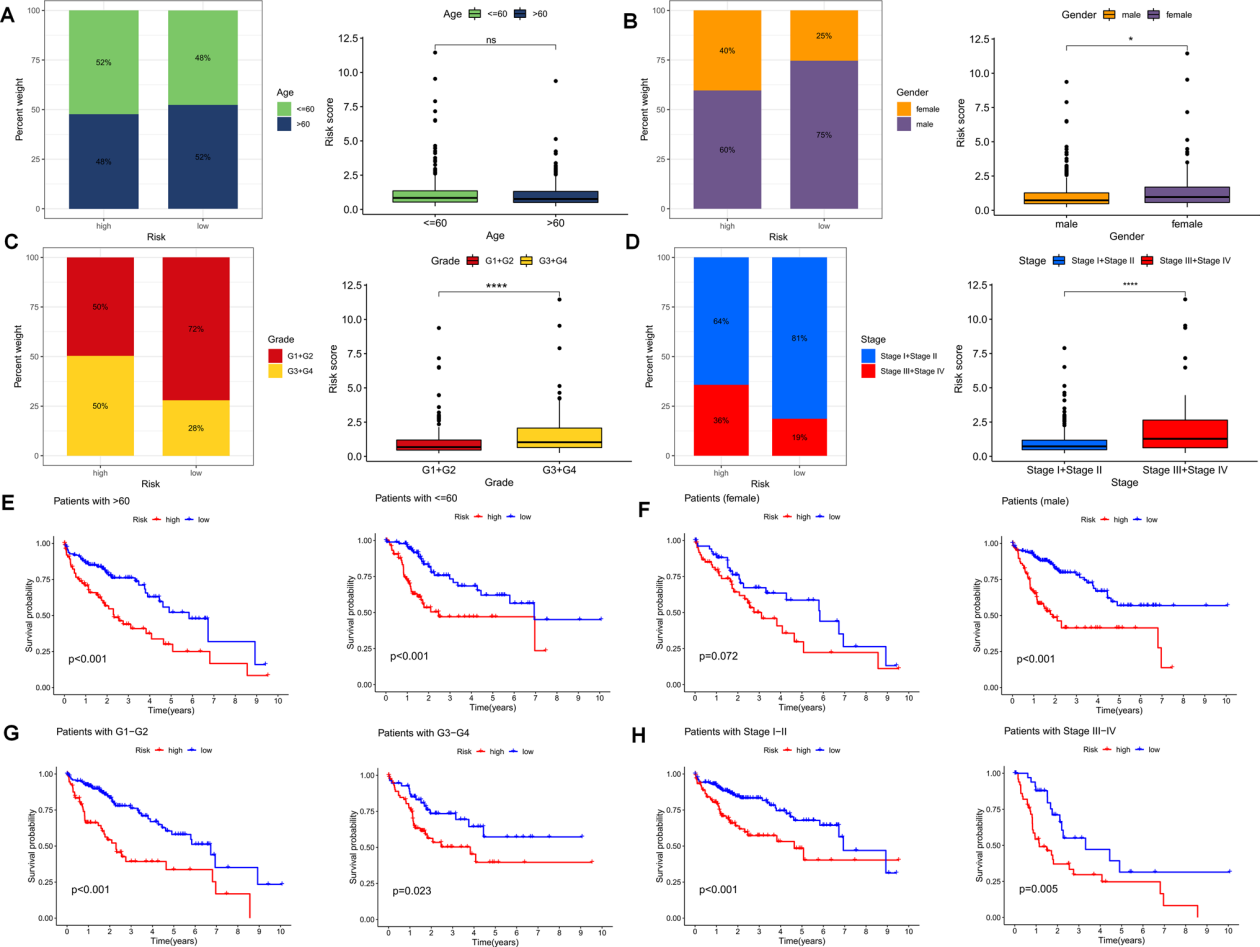
Risk scores and clinical histological characteristics. Differences in risk scores between groups with different clinical characteristics in the TCGA cohort. **A** age, **B** sex, **C** grade, and **D** stage. Survival curves of high- and low-risk score groups in different clinical characteristics groups. **E** Age, **F** sex, **G** grade, and **H** stage. ns, no significance, *p < 0.05, **p < 0.01, ***p < 0.001, and ****p < 0.0001

The frequency of distribution of the four FCRLs among different characteristics was found to vary, with higher risk scores observed in female patients and those with higher tumor grade and stage (*p* < 0.05, Fig. 5A–D). The samples were then grouped based on age (whether older than 60 years), gender (male versus female), tumor grade (I or II and III or IV), pathological stage (I, II and III, IV), and T stage (T1–2 and T3–4) to evaluate the OS of patients in different subgroups (Fig. 5E–H). It was observed that in almost all subgroups, HR patients had significantly lower overall survival than LR patients, indicating that our risk model can accurately predict the prognosis of LIHC patients in different clinical subgroups.

### Comparison of PCA analysis and construction of nomogram

**Fig. 6:**
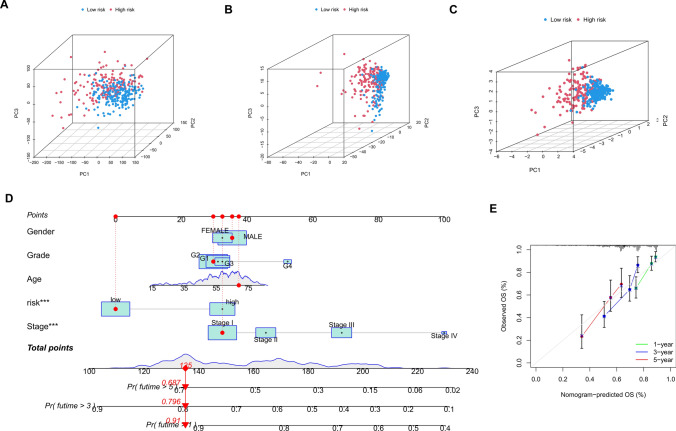
PCA analysis and construction of nomogram. **A** PCA plots were drawn for all genes in the TCGA cohort. **B** PCA maps drawn with all FCRLs involved. **C** PCA maps using the FCRL signature. **D** A nomogram indicating the risk groups and clinical features that forecast survival for one, three, and five years. **E** Calibration graphs are used to assess concordance between expected and actual results after one, three, and five years

PCA was performed on the complete transcriptome of TCGA, all FCRLs, and lncRNAs in the risk model, revealing significant differences in the transcriptional profiles between the high-risk (HR) and low-risk (LR) groups. Furthermore, lncRNAs in the FCRL model were found to be more effective in classifying patients into different risk groups, as demonstrated in Fig. 6A–C. Our approach thus proves to be effective in distinguishing LR and HR populations, taking into account various factors such as age, gender, grade, stage, and FCRL risk score, which were used to develop the nomogram (Fig. 6D). To evaluate the accuracy of the prognostic model, calibration plots were drawn, showing good agreement between the expected overall survival (OS) and the actual OS of liver cancer patients, as depicted in Fig. 6E.

### Immune microenvironment and immune cell infiltration

**Fig. 7:**
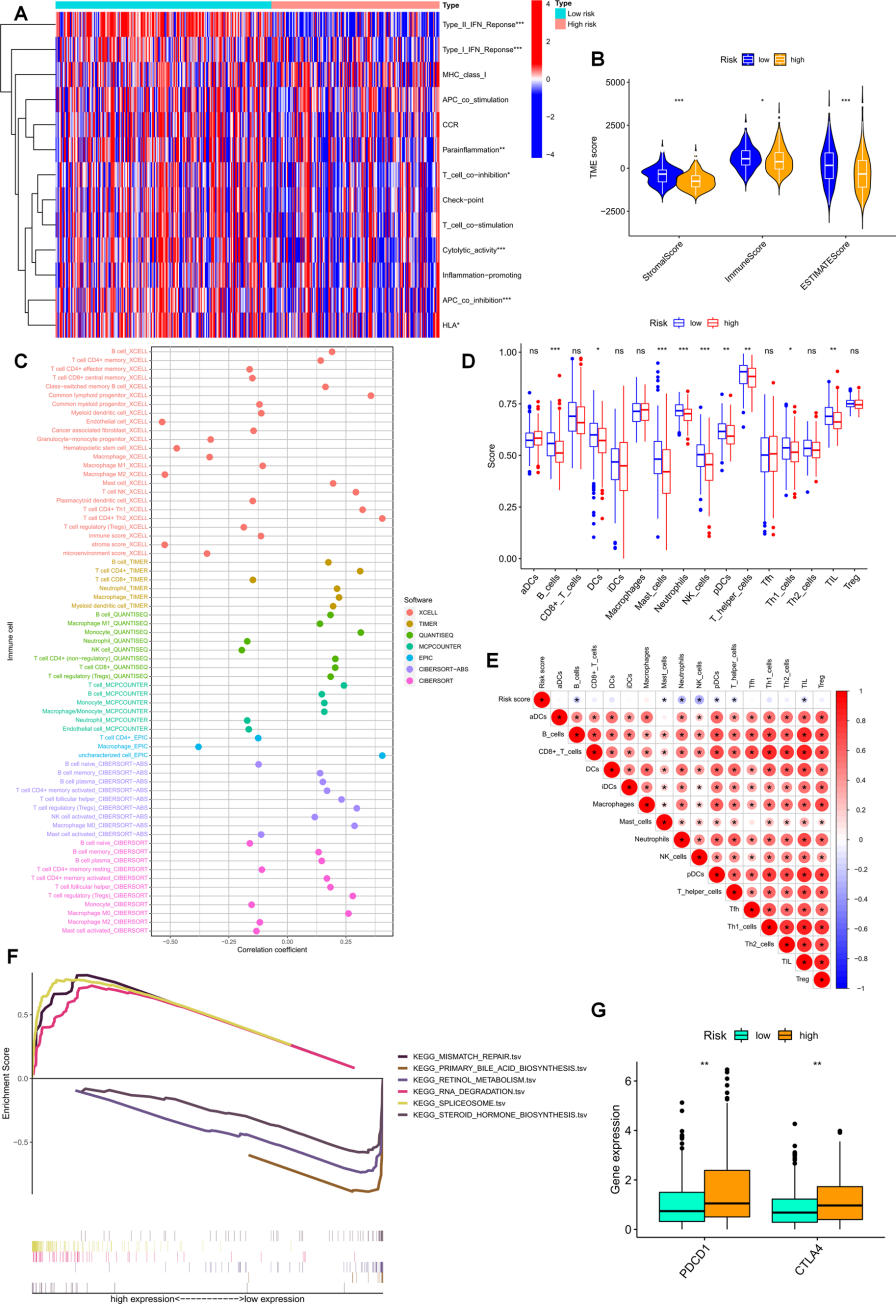
Risk scores of FCRLs predict tumor microenvironment and immune function. **A** Comparison of immune function between high-risk and low-risk populations. **B** Comparison of TME scores between high-risk and low-risk groups. **C** Correlation between risk scores and immune cell content shown in bubble plots generated by different algorithms. **D** Differences in immune cell enrichment scores between the high-risk and low-risk groups. **E** Correlation between risk scores and immune cell enrichment scores. **F** GSEA showing different enrichment levels of the KEGG pathway. **G** Comparison of PD-1 and CTLA-4 expression levels in the two risk groups. ns: no significance, *p < 0.05, **p < 0.01, and ***p < 0.001

In this study, the ssGSEA algorithm was used to measure the enrichment scores of various immune cell subtypes and immune-related pathways, and further investigate the relationship between risk scores and immune cells and functions. The results showed significant correlations between risk scores and B cells, DCs, mast cells, neutrophils, NK cells, pDCs, helper T cells, Th1 cells, and tumor-infiltrating lymphocytes (TILs) (Fig. 7D and E). The levels of all immunological pathways were higher in the LR group than in the HR group (Fig. 7A). Through multiple algorithm analyses, we found that the constructed FCRL risk score was related to the abundance of immune cells in the tumor microenvironment. For example, the results of CIBERSORT, XCELL, and TIMER indicated a significant negative correlation between macrophage content and risk score (Fig. 7C). Tumor-infiltrating immune cells (TIICs) play a very important role in the tumor microenvironment, not only participating in tumor immune surveillance and clearance but also regulating biological processes such as tumor growth, invasion, and metastasis by interacting with tumor cells. And tumor microenvironment can provide support and protection for tumor development, as well as an important factor in resistance to immunotherapy. Therefore, an in-depth study of the mechanisms of interaction between tumor-infiltrating immune cells and tumor microenvironment can help us better understand the process of tumorigenesis and development and provide a theoretical and practical basis for the development of new tumor treatment strategies. TME scores obtained from the ESTIMATE package showed that patients in the LR group had higher TME scores (Fig. 7B). We compared the expression differences of two common immune checkpoint proteins, PD-1 and CTLA-4, between the HR and LR groups in liver cancer and found that their expression was higher in the HR group (Fig. 7G). Additionally, we used GSEA to study the potential biological functional differences between HR and LR patients and selected the top six enriched signaling pathways. For example, the HR group is closely related to the mismatch repair pathway (Fig. 7F).

### Prediction of the effect of immunotherapy and chemotherapy

**Fig. 8:**
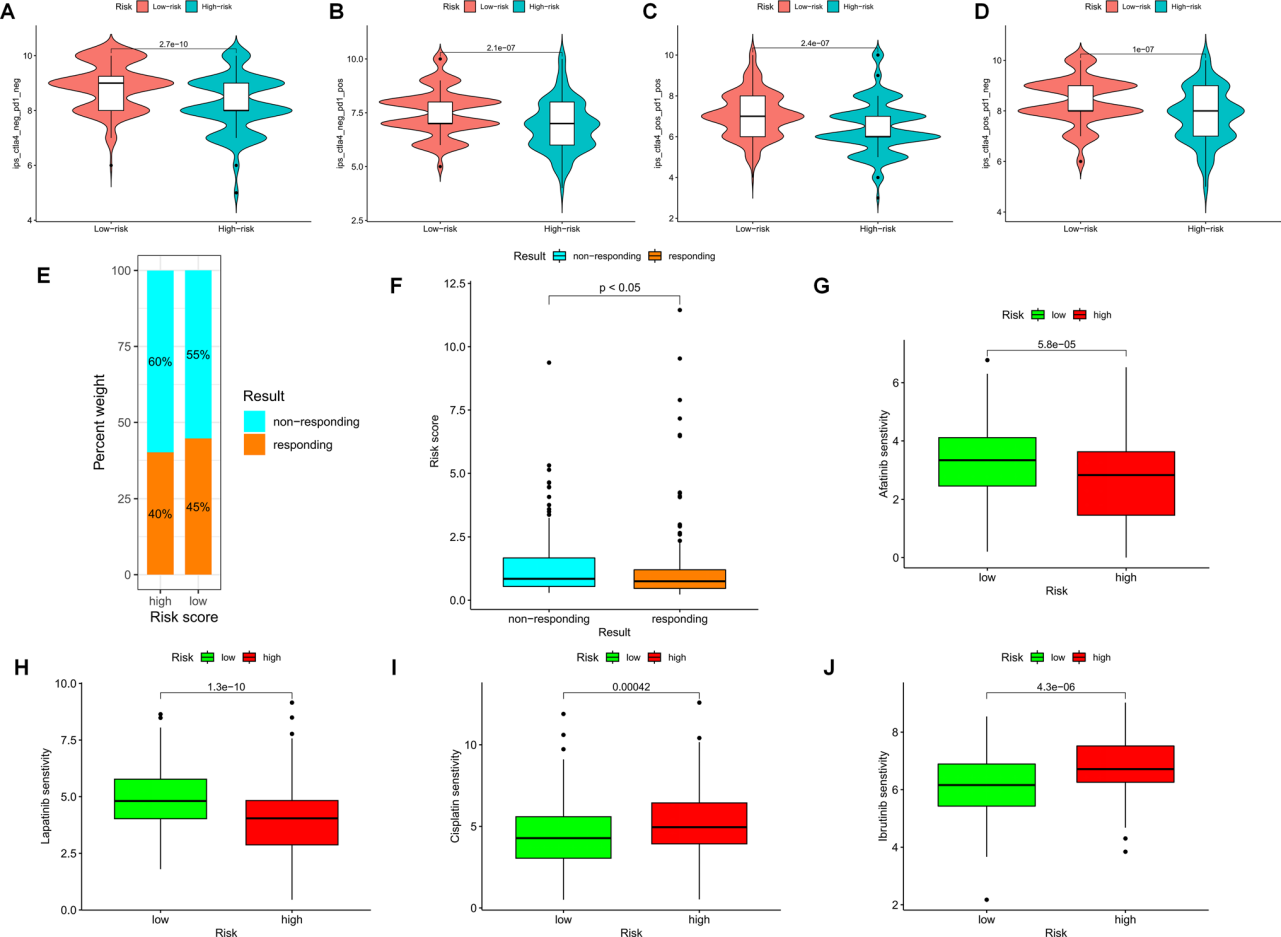
Prediction of immunotherapy and chemotherapy effects. **A**–**D** Distribution of immune scores (IPS) between high-risk and low-risk groups. **E** and **F** Four-FCRL signatures predict immunotherapy response outcomes in hepatocellular carcinoma patients. **G**–**J** IC50 values for afatinib, lapatinib, cisplatin, and ibrutinib in high-risk and low-risk groups, assessing chemotherapeutic agent sensitivity

Higher immune scores (IPSs) were associated with a stronger response to PD-1 and CTLA-4 blockers, as shown in the violin plot (Fig. 8A–D). Patients with lower risk scores were found to be more likely to benefit from immunotherapy, as demonstrated by results obtained through the immuneAI portal (Fig. 8E and F). The "pRRophetic" R package was used to investigate the potential sensitivity of clinical agents in both the HR and LR groups. Afatinib and ibrutinib, which are commonly used to treat hepatocellular carcinomas, showed a higher IC50 in patients in the HR group, while lapatinib and cisplatin showed a higher IC50 in LR patients (Fig. 8G–J).

### Validation of model genes

**Fig. 9:**
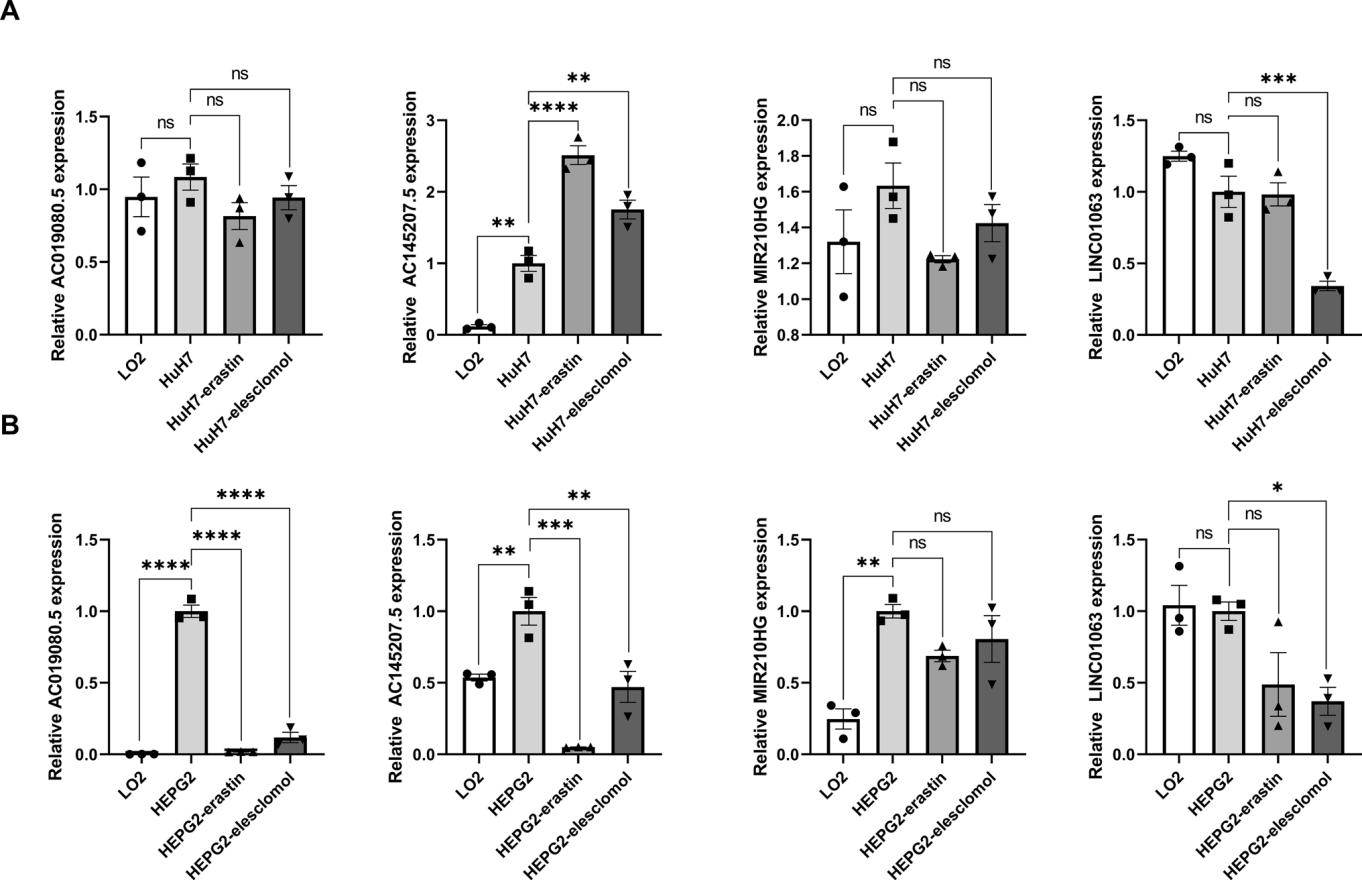
qRT-PCR assay in cuproptosis and ferroptosis models. **A** and **B** Expression levels of four FRCLs in normal hepatocytes and hepatoma cells according to qRT-PCR. Four FRCLs expression levels in hepatoma cells and after addition of erastin and elesclomol-2-CuCl2. ns, no significance, *p < 0.05, **p < 0.01, ***p < 0.001, and ****p < 0.0001

Finally, the expression of these FCRLs was examined by qRT-PCR in HuH7 and HEPG2 cells after the addition of elesclomol-2-CuCl2 and erastin. It was found that the expression of all four FCRLs differed significantly between normal hepatocytes and hepatoma cells. Specifically, the expression of these FCRLs was significantly reduced after the addition of ferroptosis and cuproptosis inducers (Fig. 9A and B). These results suggest that FCRLs have a differential expression pattern in normal and cancerous liver cells, potentially playing a crucial role in the processes of ferroptosis and cuproptosis. The significant reduction in FCRL expression upon treatment with these inducers highlights their potential as therapeutic targets in hepatocellular carcinoma. This discovery provides a basis for developing new therapeutic strategies by modulating FCRLs and offers insights into their mechanisms in cell death processes.

## Discussion

The prevalence of HCC is a concerning issue globally, with 830,000 liver cancer deaths reported in 2020, of which China accounted for 47.1%. The high mortality rate is largely due to the lack of effective early diagnostic measures. In China, 71% of liver cancer patients are only diagnosed in the middle to late stages, resulting in missed opportunities for radical surgical treatment [46, 47]. Given the poor prognosis of hepatocellular carcinoma patients, it is imperative to find new and promising biomarkers and predictors for better patient outcomes [48, 49]. Traditional bioinformatics studies have mainly focused on single biomarkers or biological processes. However, using combined models that incorporate multiple linked genes with diverse biological properties could lead to more accurate prognostic predictions and tailored tumor treatment.

Programmed cell death, like the natural shedding of leaves or flowers, is a natural process among cells. When it occurs, apoptotic cells are disposed of among normal tissue cells without causing inflammation or scarring [50]. But when this process is disrupted, it can lead to uncontrolled growth of malignant cells, such as cancer. Recently, ferroptosis and cuproptosis have become the focus of much attention in the cancer community due to their unique forms of cell death. They differ from other types of regulated cell death, such as apoptosis, and thus hold great promise for cancer therapy [6, 51]. Studies have found that ferroptosis plays a crucial role in HCC treatment through immunotherapy and radiation [52]. Additionally, gene signatures related to ferroptosis and cuproptosis are increasingly being identified to predict prognosis and immune efficacy in HCC patients [53, 54]. Despite this, the prognostic significance of ferroptosis and cuproptosis-related lncRNAs (FCRLs) in HCC is still unknown. Therefore, investigating FCRLs is essential for early detection and treatment planning in HCC, which could ultimately improve patient survival.

Utilizing the GSVA method, we assessed the enrichment of ferroptosis and cuproptosis pathways in samples from the TCGA cohort to elucidate the potential role of FCRLs in HCC patients. Our findings indicated that the WGCNA algorithm was effective in identifying FCRLs and accurately predicting patient survival. To construct a prognostic signature comprising four FCRLs (AC019080.5, AC145207.5, MIR210HG, and LINC01063) for delineating the immunological landscape and prognostic profile of HCC patients, we employed LASSO regression and Cox proportional hazards regression analyses. Validation analyses corroborated the efficacy of the FCRL risk score model in prognostication for HCC patients. The identified risk signatures associated with ferroptosis and cuproptosis were significantly correlated with patient prognosis and immune landscape in HCC, suggesting a potential role of these four risk genes in modulating the tumor microenvironment. These findings highlight the critical importance of these biological processes and their associated genes in the pathogenesis of HCC and underscore their potential as therapeutic targets.

The four risk genes have been extensively studied in various types of cancer. AC019080.5 has been identified as an immune-related lncRNA affecting the prognosis of endometrial cancer (UCEC) and may be a useful therapeutic target and molecular biomarker for UCEC [55]. While Liu suggested that AC019080.5 is a potential prognostic biomarker associated with UCEC autophagy [55]. AC145207.5, another risk gene, has been extensively studied in the context of hepatocellular carcinoma and is believed to be involved in immune-related pathways such as glycolysis, inflammatory response, and pyroptosis [56–58]. The overexpression of MIR210HG has been linked to unfavorable clinical outcomes in several cancer types, including glioma, endometrial cancer, and lung cancer [59–61]. Silencing MIR210HG has been shown to effectively inhibit the proliferation, migration, and invasion of hepatocellular carcinoma cells [62]. Lastly, LINC01063 has been implicated in various tumor cell processes, including proliferation, migration, invasion, and epithelial–mesenchymal transition (EMT). Its involvement in these mechanisms suggests its potential as a key regulator of cancer progression and metastasis [63, 64]. Zhang et al. suggested that LINC01063 was linked to HCC ferroptosis and impacted patient survival [65], and it is also thought to be involved in autophagy and cuproptosis in different types of tumors [66, 67].

By examining the expression levels of four FCRLs identified through screening, a risk score was developed in this study. Patients were then divided into two groups based on their risk score, with those in the high-risk (HR) group having a worse prognosis compared to the low-risk (LR) group. The efficacy of the risk score model was assessed using various statistical analyses, including the C-index, ROC curve, and Cox regression analysis. The results of these analyses suggest that the risk score model has the potential as a valuable standalone predictor for HCC patients. These findings provide important insights into the development of personalized treatment strategies for HCC patients, as the risk score model may help identify individuals who require more intensive or aggressive treatment approaches. Additionally, this study highlights the importance of incorporating biomarkers such as FCRLs into clinical practice to improve prognostic accuracy and optimize patient outcomes. Higher risk scores were associated with higher tumor grades, which can be visualized through column line plots incorporating clinicopathological components. While age, gender, and tumor grade are common clinical indicators for predicting HCC prognosis, our model demonstrated greater net return and potential impact on clinical decision-making. Using the nomogram generated from the model, physicians can personalize anti-tumor treatments for individual patients.

Immunosuppressive cells, immune effector cells, the cytokine environment, and the intrinsic signaling pathways of tumor cells interact to form the HCC immune microenvironment. Their various etiologies affect the immune response and produce distinctive microenvironmental characteristics. The LR group has an abundance of immune-related biological processes and pathways, likely due to distinct antigens produced by iron and copper-loving cells [68]. We examined the immune cell landscape and associated immunological pathways in each risk group and discovered that tumor-infiltrating T lymphocytes (TIL) and natural killer cells (NK) were more prevalent in the LR group. TIL are tumor-specific lymphocytes that are extracted from tumor tissue, cultured and expanded in vitro, and then infused back into patients, and have a better killing effect on tumor cells [69]. Because it recognizes multiple antigens, TIL may have higher anti-tumor efficiency and lower toxicity than treatments that target a single antigen in tumor cells [70]. Numerous clinical trials have demonstrated the potential of TIL (tumor-infiltrating lymphocytes) therapy to increase recurrence-free survival and overall survival rates among liver cancer patients, while also reducing the likelihood of cancer recurrence [69]. In HCC immune surveillance, natural immune effector cells such as natural killer cells are crucial [71]. TP53 mutations occur in approximately 40% of HCCs and are more prevalent in non-inflammatory HCCs. We found that TP53 functional deficiency in the HR group contributed to immunosuppressive cell recruitment. Finally, overexpression of the immune checkpoint molecules PD-1 and CTLA-4 in the HR group often led to T cell failure and a worse prognosis [72]. While immunotherapy shows great promise as an alternative treatment for HCC, it is important to note that patients may not respond equally to this approach. In fact, some patients may not respond at all, highlighting the need for further research to develop more effective and personalized therapies. LR group patients responded better to ICB immunotherapy, according to both immuneAI and TCIA algorithms. However, generating an effective response to immunotherapy remains challenging for the significantly immunosuppressed TME in the HR group. Therefore, our model suggests that higher risk scores are related to poorer anti-tumor immunity. Nevertheless, the nomogram can be utilized to personalize anti-tumor treatments based on each patient's risk score and other clinical indicators.

In summation, we have created a new predictive signature that combines cuproptosis and ferroptosis in liver cancer. This signature can not only give an accurate forecast of HCC patient prognosis but also help to predict key factors such as immune function and the tumor microenvironment. This signature can also inform drug and immunotherapy decisions for HCC patients. This is the first time lncRNAs have been linked to both cuproptosis and ferroptosis in HCC, and our findings offer new insights into the roles these two forms of cell death play in the disease.

## Supplementary Information

Below is the link to the electronic supplementary material.

Supplementary file1 (DOC 551 KB)

## Data Availability

The datasets analyzed in the current study are available in the TCGA repository (http://cancergenome.nih.gov/) and GEO (https://www.ncbi.nlm.nih.gov/geo/).

## References

[1] Rebouissou S, Nault JC. Advances in molecular classification and precision oncology in hepatocellular carcinoma. J Hepatol. 2020;72(2):215–29. 31954487 10.1016/j.jhep.2019.08.017

[2] Bray F, et al. Global cancer statistics 2018: GLOBOCAN estimates of incidence and mortality worldwide for 36 cancers in 185 countries. CA Cancer J Clin. 2018;68(6):394–424. 30207593 10.3322/caac.21492

[3] Singal AG, Lampertico P, Nahon P. Epidemiology and surveillance for hepatocellular carcinoma: new trends. J Hepatol. 2020;72(2):250–61. 31954490 10.1016/j.jhep.2019.08.025 PMC6986771

[4] Pais R, et al. NAFLD and liver transplantation: current burden and expected challenges. J Hepatol. 2016;65(6):1245–57. 27486010 10.1016/j.jhep.2016.07.033 PMC5326676

[5] Pinter M, Scheiner B, Peck-Radosavljevic M. Immunotherapy for advanced hepatocellular carcinoma: a focus on special subgroups. Gut. 2021;70(1):204–14. 32747413 10.1136/gutjnl-2020-321702 PMC7788203

[6] Lei G, Zhuang L, Gan B. Targeting ferroptosis as a vulnerability in cancer. Nat Rev Cancer. 2022;22(7):381–96. 35338310 10.1038/s41568-022-00459-0 PMC10243716

[7] Yan Z, et al. ACLY promotes gastric tumorigenesis and accelerates peritoneal metastasis of gastric cancer regulated by HIF-1A. Cell Cycle. 2023;22(20):2288–301. 38009671 10.1080/15384101.2023.2286805 PMC10730177

[8] Xiao J, et al. Obesity promotes lipid accumulation in lymph node metastasis of gastric cancer: a retrospective case-control study. Lipids Health Dis. 2022;21(1):123. 36397145 10.1186/s12944-022-01734-7 PMC9673345

[9] Liu K, et al. Peritoneal high-fat environment promotes peritoneal metastasis of gastric cancer cells through activation of NSUN2-mediated ORAI2 m5C modification. Oncogene. 2023;42(24):1980–93. 37130916 10.1038/s41388-023-02707-5

[10] Liu K, et al. Unveiling the oncogenic role of CLDN11-secreting fibroblasts in gastric cancer peritoneal metastasis through single-cell sequencing and experimental approaches. Int Immunopharmacol. 2024;129: 111647. 38335659 10.1016/j.intimp.2024.111647

[11] Fang X, et al. Cancer associated fibroblasts-derived SULF1 promotes gastric cancer metastasis and CDDP resistance through the TGFBR3-mediated TGF-β signaling pathway. Cell Death Discov. 2024;10(1):111. 38438372 10.1038/s41420-024-01882-y PMC10912303

[12] Liu J, et al. Deciphering drug resistance in gastric cancer: potential mechanisms and future perspectives. Biomed Pharmacother. 2024;173: 116310. 38394851 10.1016/j.biopha.2024.116310

[13] Huang L, et al. Intracellular amyloid toxicity induces oxytosis/ferroptosis regulated cell death. Cell Death Dis. 2020;11(10):828. 33024077 10.1038/s41419-020-03020-9 PMC7538552

[14] Stockwell BR, Jiang X, Gu W. Emerging mechanisms and disease relevance of ferroptosis. Trends Cell Biol. 2020;30(6):478–90. 32413317 10.1016/j.tcb.2020.02.009 PMC7230071

[15] Ma X, et al. CD36-mediated ferroptosis dampens intratumoral CD8(+) T cell effector function and impairs their antitumor ability. Cell Metab. 2021;33(5):1001-1012e5. 33691090 10.1016/j.cmet.2021.02.015 PMC8102368

[16] Wang W, et al. CD8(+) T cells regulate tumour ferroptosis during cancer immunotherapy. Nature. 2019;569(7755):270–4. 31043744 10.1038/s41586-019-1170-y PMC6533917

[17] Lv H, et al. Comprehensive analysis of cuproptosis-related genes in immune infiltration and prognosis in melanoma. Front Pharmacol. 2022;13: 930041. 35837286 10.3389/fphar.2022.930041 PMC9273972

[18] Tsvetkov P, et al. Copper induces cell death by targeting lipoylated TCA cycle proteins. Science. 2022;375(6586):1254–61. 35298263 10.1126/science.abf0529 PMC9273333

[19] Tang D, Chen X, Kroemer G. Cuproptosis: a copper-triggered modality of mitochondrial cell death. Cell Res. 2022;32(5):417–8. 35354936 10.1038/s41422-022-00653-7 PMC9061796

[20] Blockhuys S, et al. Defining the human copper proteome and analysis of its expression variation in cancers. Metallomics. 2017;9(2):112–23. 27942658 10.1039/c6mt00202a

[21] Brady DC, et al. Copper chelation inhibits BRAF(V600E)-driven melanomagenesis and counters resistance to BRAF(V600E) and MEK1/2 inhibitors. Cancer Res. 2017;77(22):6240–52. 28986383 10.1158/0008-5472.CAN-16-1190 PMC5690876

[22] Davis CI, et al. Altered copper homeostasis underlies sensitivity of hepatocellular carcinoma to copper chelation. Metallomics. 2020;12(12):1995–2008. 33146201 10.1039/d0mt00156b PMC8315290

[23] Zhao XY, Lin JD. Long noncoding RNAs: a new regulatory code in metabolic control. Trends Biochem Sci. 2015;40(10):586–96. 26410599 10.1016/j.tibs.2015.08.002 PMC4584418

[24] Slack FJ, Chinnaiyan AM. The role of non-coding RNAs in oncology. Cell. 2019;179(5):1033–55. 31730848 10.1016/j.cell.2019.10.017 PMC7347159

[25] Xia A, et al. The cancer-testis lncRNA lnc-CTHCC promotes hepatocellular carcinogenesis by binding hnRNP K and activating YAP1 transcription. Nat Cancer. 2022;3(2):203–18. 35122073 10.1038/s43018-021-00315-4

[26] Xu Z, et al. Construction of a ferroptosis-related nine-lncRNA signature for predicting prognosis and immune response in hepatocellular carcinoma. Front Immunol. 2021;12: 719175. 34603293 10.3389/fimmu.2021.719175 PMC8484522

[27] Zhou N, Bao J. FerrDb: a manually curated resource for regulators and markers of ferroptosis and ferroptosis-disease associations. Database (Oxford). 2020;baaa021. 10.1093/database/baaa021 PMC7100629 32219413

[28] Hänzelmann S, Castelo R, Guinney J. GSVA: gene set variation analysis for microarray and RNA-seq data. BMC Bioinf. 2013;14:7. 10.1186/1471-2105-14-7 PMC3618321 23323831

[29] Zhang P, et al. Purine metabolism in lung adenocarcinoma: a single-cell analysis revealing prognostic and immunotherapeutic insights. J Cell Mol Med. 2024;28(8): e18284. 38597415 10.1111/jcmm.18284 PMC11005461

[30] Langfelder P, Horvath S. WGCNA: an R package for weighted correlation network analysis. BMC Bioinf. 2008;9:559. 10.1186/1471-2105-9-559 PMC2631488 19114008

[31] Ritchie ME, et al. limma powers differential expression analyses for RNA-sequencing and microarray studies. Nucleic Acids Res. 2015;43(7): e47. 25605792 10.1093/nar/gkv007 PMC4402510

[32] Subramanian A, et al. Gene set enrichment analysis: a knowledge-based approach for interpreting genome-wide expression profiles. Proc Natl Acad Sci U S A. 2005;102(43):15545–50. 16199517 10.1073/pnas.0506580102 PMC1239896

[33] Zhang P, et al. Clinical prognostication and immunotherapy response prediction in esophageal squamous cell carcinoma using the DNA damage repair-associated signature. Environ Toxicol. 2024;39(5):2803–16. 38287713 10.1002/tox.24155

[34] Zhang P, et al. Mast cell marker gene signature: prognosis and immunotherapy response prediction in lung adenocarcinoma through integrated scRNA-seq and bulk RNA-seq. Front Immunol. 2023;14:1189520. 37256127 10.3389/fimmu.2023.1189520 PMC10225553

[35] Yoshihara K, et al. Inferring tumour purity and stromal and immune cell admixture from expression data. Nat Commun. 2013;4:2612. 24113773 10.1038/ncomms3612 PMC3826632

[36] Aran D, Hu Z, Butte AJ. xCell: digitally portraying the tissue cellular heterogeneity landscape. Genome Biol. 2017;18(1):220. 29141660 10.1186/s13059-017-1349-1 PMC5688663

[37] Li T, et al. TIMER2.0 for analysis of tumor-infiltrating immune cells. Nucleic Acids Res. 2020;48(W1):W509–14. 32442275 10.1093/nar/gkaa407 PMC7319575

[38] Racle J, et al. Simultaneous enumeration of cancer and immune cell types from bulk tumor gene expression data. Elife. 2017;6:e26476. 29130882 10.7554/eLife.26476 PMC5718706

[39] Chen B, et al. Profiling tumor infiltrating immune cells with CIBERSORT. Methods Mol Biol. 2018;1711:243–59. 29344893 10.1007/978-1-4939-7493-1_12 PMC5895181

[40] Charoentong P, et al. Pan-cancer immunogenomic analyses reveal genotype-immunophenotype relationships and predictors of response to checkpoint blockade. Cell Rep. 2017;18(1):248–62. 28052254 10.1016/j.celrep.2016.12.019

[41] Miao YR, et al. ImmuCellAI: a unique method for comprehensive T-cell subsets abundance prediction and its application in cancer immunotherapy. Adv Sci (Weinh). 2020;7(7):1902880. 32274301 10.1002/advs.201902880 PMC7141005

[42] Geeleher P, Cox N, Huang RS. pRRophetic: an R package for prediction of clinical chemotherapeutic response from tumor gene expression levels. PLoS ONE. 2014;9(9): e107468. 25229481 10.1371/journal.pone.0107468 PMC4167990

[43] Yuan Q, et al. Low-density lipoprotein receptor promotes crosstalk between cell stemness and tumor immune microenvironment in breast cancer: a large data-based multi-omics study. J Transl Med. 2023;21(1):871. 38037058 10.1186/s12967-023-04699-y PMC10691045

[44] Geeleher P, Cox NJ, Huang RS. Clinical drug response can be predicted using baseline gene expression levels and in vitro drug sensitivity in cell lines. Genome Biol. 2014;15(3):R47. 24580837 10.1186/gb-2014-15-3-r47 PMC4054092

[45] Zhang G, Sun J, Zhang X. A novel Cuproptosis-related LncRNA signature to predict prognosis in hepatocellular carcinoma. Sci Rep. 2022;12(1):11325. 35790864 10.1038/s41598-022-15251-1 PMC9256635

[46] Dixon SJ, et al. Ferroptosis: an iron-dependent form of nonapoptotic cell death. Cell. 2012;149(5):1060–72. 22632970 10.1016/j.cell.2012.03.042 PMC3367386

[47] Zhang P et al. Unraveling the role of low-density lipoprotein-related genes in lung adenocarcinoma: insights into tumor microenvironment and clinical prognosis. Environ Toxicol. 2024;39(3):1045–888. 10.1002/tox.24230 38488684

[48] Bouattour M, et al. Recent developments of c-Met as a therapeutic target in hepatocellular carcinoma. Hepatology. 2018;67(3):1132–49. 28862760 10.1002/hep.29496 PMC5873445

[49] Song D, et al. Identification of an endoplasmic reticulum stress-related gene signature to evaluate the immune status and predict the prognosis of hepatocellular carcinoma. Front Genet. 2022;13: 850200. 35711939 10.3389/fgene.2022.850200 PMC9197218

[50] Lai G, et al. Development of a hallmark pathway-related gene signature associated with immune response for lower grade gliomas. Int J Mol Sci. 2022;23(19):11971. 36233273 10.3390/ijms231911971 PMC9570050

[51] Chen Y, et al. Elevated expression of PDZD11 is associated with poor prognosis and immune infiltrates in hepatocellular carcinoma. Front Genet. 2021;12: 669928. 34093661 10.3389/fgene.2021.669928 PMC8176286

[52] Elmore S. Apoptosis: a review of programmed cell death. Toxicol Pathol. 2007;35(4):495–516. 17562483 10.1080/01926230701320337 PMC2117903

[53] Hu H, et al. New anti-cancer explorations based on metal ions. J Nanobiotechnology. 2022;20(1):457. 36274142 10.1186/s12951-022-01661-w PMC9590139

[54] Lang X, et al. Radiotherapy and immunotherapy promote tumoral lipid oxidation and ferroptosis via synergistic repression of SLC7A11. Cancer Discov. 2019;9(12):1673–85. 31554642 10.1158/2159-8290.CD-19-0338 PMC6891128

[55] Wang L, et al. Identification of a ferroptosis-related long noncoding RNA prognostic signature and its predictive ability to immunotherapy in hepatocellular carcinoma. Front Genet. 2021;12: 682082. 34745200 10.3389/fgene.2021.682082 PMC8566703

[56] Huang EM, et al. Cuproptosis-related long non-coding RNAs model that effectively predicts prognosis in hepatocellular carcinoma. World J Gastrointest Oncol. 2022;14(10):1981–2003. 36310708 10.4251/wjgo.v14.i10.1981 PMC9611437

[57] Wang Z, et al. An immune-related long noncoding RNA signature as a prognostic biomarker for human endometrial cancer. J Oncol. 2021;2021:9972454. 34925511 10.1155/2021/9972454 PMC8683168

[58] Liu H, Cheng Y. Identification of autophagy-related long non-coding RNAs in endometrial cancer via comprehensive bioinformatics analysis. BMC Womens Health. 2022;22(1):85. 35321716 10.1186/s12905-022-01667-4 PMC8943986

[59] Xia X, et al. Identification of glycolysis-related lncRNAs and the novel lncRNA WAC-AS1 promotes glycolysis and tumor progression in hepatocellular carcinoma. Front Oncol. 2021;11: 733595. 34527595 10.3389/fonc.2021.733595 PMC8437343

[60] Li X, et al. Inflammatory response-related long non-coding RNA signature predicts the prognosis of hepatocellular carcinoma. J Oncol. 2022;2022:9917244. 35342418 10.1155/2022/9917244 PMC8947866

[61] Cheng Z, et al. Prognostic pyroptosis-related lncRNA signature predicts the efficacy of immunotherapy in hepatocellular carcinoma. Biochem Biophys Rep. 2022;32: 101389. 36438599 10.1016/j.bbrep.2022.101389 PMC9684700

[62] Min W, et al. Long noncoding RNA miR210HG as a potential biomarker for the diagnosis of glioma. PLoS ONE. 2016;11(9): e0160451. 27673330 10.1371/journal.pone.0160451 PMC5038942

[63] Ma J, et al. lncRNA MIR210HG promotes the progression of endometrial cancer by sponging miR-337-3p/137 via the HMGA2-TGF-beta/Wnt pathway. Mol Ther Nucleic Acids. 2021;24:905–22. 34094710 10.1016/j.omtn.2021.04.011 PMC8141672

[64] Yu T, et al. MIR210HG regulates glycolysis, cell proliferation, and metastasis of pancreatic cancer cells through miR-125b-5p/HK2/PKM2 axis. RNA Biol. 2021;18(12):2513–30. 34110962 10.1080/15476286.2021.1930755 PMC8632125

[65] Wang Y, et al. MIR210HG predicts poor prognosis and functions as an oncogenic lncRNA in hepatocellular carcinoma. Biomed Pharmacother. 2019;111:1297–301. 30841443 10.1016/j.biopha.2018.12.134

[66] Ghafouri-Fard S, et al. Down-regulation of a panel of immune-related lncRNAs in breast cancer. Pathol Res Pract. 2021;224: 153534. 34175685 10.1016/j.prp.2021.153534

[67] Xu J, et al. LINC01063 functions as an oncogene in melanoma through regulation of miR-5194-mediated SOX12 expression. Melanoma Res. 2022;32(4):218–30. 35256570 10.1097/CMR.0000000000000803

[68] Zhang Z, et al. Construction and validation of a ferroptosis-related lncRNA signature as a novel biomarker for prognosis, immunotherapy and targeted therapy in hepatocellular carcinoma. Front Cell Dev Biol. 2022;10: 792676. 35295858 10.3389/fcell.2022.792676 PMC8919262

[69] Huang Q, et al. Prognostic prediction of head and neck squamous cell carcinoma: construction of cuproptosis-related long non-coding RNA signature. J Clin Lab Anal. 2022;36(11): e24723. 36189780 10.1002/jcla.24723 PMC9701877

[70] Cheng L, et al. Identification and validation of six autophagy-related Long non-coding RNAs as prognostic signature in colorectal cancer. Int J Med Sci. 2021;18(1):88–98. 33390777 10.7150/ijms.49449 PMC7738973

[71] Friedmann Angeli JP, Krysko DV, Conrad M. Ferroptosis at the crossroads of cancer-acquired drug resistance and immune evasion. Nat Rev Cancer. 2019;19(7):405–14. 31101865 10.1038/s41568-019-0149-1

[72] Zhang L, et al. Immunotherapy for advanced hepatocellular carcinoma, where are we? Biochim Biophys Acta Rev Cancer. 2020;1874(2): 188441. 33007432 10.1016/j.bbcan.2020.188441

[73] Jiang SS, et al. A phase I clinical trial utilizing autologous tumor-infiltrating lymphocytes in patients with primary hepatocellular carcinoma. Oncotarget. 2015;6(38):41339–49. 26515587 10.18632/oncotarget.5463 PMC4747409

[74] Llovet JM, et al. Immunotherapies for hepatocellular carcinoma. Nat Rev Clin Oncol. 2022;19(3):151–72. 34764464 10.1038/s41571-021-00573-2

[75] Xu F, et al. Immune checkpoint therapy in liver cancer. J Exp Clin Cancer Res. 2018;37(1):110. 29843754 10.1186/s13046-018-0777-4 PMC5975687

