# Supplementary material for: Long non-coding RNAs in ferroptosis and cuproptosis impact on prognosis and treatment in hepatocellular carcinoma
Source: Clin Exp Med. 2024 Jun 22;24(1):135. doi: 10.1007/s10238-024-01397-x (PMC11193701; doi:10.1007/s10238-024-01397-x)
Supplement: Supplementary file 1 — Supplementary file1 (DOC 551 KB) [file 10238_2024_1397_MOESM1_ESM.doc]

| **Supplementary Table 1: Sequence of primers** |
| --- |
|  |
| AC019080.5 |
| F primer (5′-3′)GCGCAATCACAGCTCACTGC |
| R primer (5′-3′)ATAGATTTTGGGGCCCGGCG |
|  |
| AC145207.5 |
| F primer (5′-3′) GACTGGCCAAGCATTTGGTG |
| R primer (5′-3′) TCTGGCCTACCTTAGGCTACAT |
|  |
| MIR210HG |
| F primer (5′-3′)CTCTGCCAGCTCCATGCCTC |
| R primer (5′-3′)CGTCACATAGGCCAGCAGGG |
|  |
| LINC01063 |
| F primer (5′-3′)TGTGCCGAGTGTGGTTTGCT |
| R primer (5′-3′)ATGATGCTCGCACCACCCAG |
|  |
| GAPDH |
| F primer (5′-3′) GGA GCG AGA TCC CTC CAA AAT |
| R primer (5′-3′)GGC TGT TGT CAT ACT TCT CATGG |

| **Supplementary Table 2: Clinical information of the patient** | | | | | |
| --- | --- | --- | --- | --- | --- |
|  |  |  |  |  |  |
| Covariates | Type | Total | Test | Train | Pvalue |
| Age | <=65 | 232(62.7%) | 110(59.46%) | 122(65.95%) | 0.237 |
| Age | >65 | 138(37.3%) | 75(40.54%) | 63(34.05%) |  |
| Gender | FEMALE | 121(32.7%) | 62(33.51%) | 59(31.89%) | 0.8246 |
| Gender | MALE | 249(67.3%) | 123(66.49%) | 126(68.11%) |  |
| Grade | G1 | 55(14.86%) | 28(15.14%) | 27(14.59%) | 0.9386 |
| Grade | G2 | 177(47.84%) | 90(48.65%) | 87(47.03%) |  |
| Grade | G3 | 121(32.7%) | 60(32.43%) | 61(32.97%) |  |
| Grade | G4 | 12(3.24%) | 5(2.7%) | 7(3.78%) |  |
| Grade | unknow | 5(1.35%) | 2(1.08%) | 3(1.62%) |  |
| Stage | Stage I | 171(46.22%) | 90(48.65%) | 81(43.78%) | 0.4974 |
| Stage | Stage II | 85(22.97%) | 41(22.16%) | 44(23.78%) |  |
| Stage | Stage III | 85(22.97%) | 42(22.7%) | 43(23.24%) |  |
| Stage | Stage IV | 5(1.35%) | 1(0.54%) | 4(2.16%) |  |
| Stage | unknow | 24(6.49%) | 11(5.95%) | 13(7.03%) |  |
| T | T1 | 181(48.92%) | 95(51.35%) | 86(46.49%) | 0.6028 |
| T | T2 | 93(25.14%) | 44(23.78%) | 49(26.49%) |  |
| T | T3 | 80(21.62%) | 37(20%) | 43(23.24%) |  |
| T | T4 | 13(3.51%) | 8(4.32%) | 5(2.7%) |  |
| T | unknow | 3(0.81%) | 1(0.54%) | 2(1.08%) |  |
| M | M0 | 266(71.89%) | 132(71.35%) | 134(72.43%) | 0.6355 |
| M | M1 | 4(1.08%) | 1(0.54%) | 3(1.62%) |  |
| M | unknow | 100(27.03%) | 52(28.11%) | 48(25.95%) |  |
| N | N0 | 252(68.11%) | 126(68.11%) | 126(68.11%) | 1 |
| N | N1 | 4(1.08%) | 2(1.08%) | 2(1.08%) |  |
| N | unknow | 114(30.81%) | 57(30.81%) | 57(30.81%) |  |

| **Supplementary Table 3: Results of univariate Cox analysis for all FCRLs** | | | | |
| --- | --- | --- | --- | --- |
| id | HR | HR.95L | HR.95H | pvalue |
| SNHG26 | 1.684558438 | 1.062196054 | 2.671575669 | 0.026663822 |
| AP003419.3 | 1.354796077 | 1.004835467 | 1.826639755 | 0.046414669 |
| AC004241.3 | 1.687730536 | 1.008950363 | 2.823166001 | 0.046161891 |
| AL807752.5 | 1.929603059 | 1.032826995 | 3.605025802 | 0.039279042 |
| LINC02870 | 1.386683728 | 1.100245597 | 1.747693212 | 0.005619435 |
| AC139100.2 | 1.395557173 | 1.003603433 | 1.940587049 | 0.047552375 |
| JMJD1C-AS1 | 1.877393682 | 1.058517508 | 3.329757903 | 0.031202376 |
| AL445524.1 | 1.255672918 | 1.042952189 | 1.511780206 | 0.016215189 |
| AL592166.1 | 2.513642184 | 1.352346135 | 4.672174429 | 0.003564644 |
| LINC00665 | 1.202895462 | 1.000644303 | 1.446025813 | 0.04920378 |
| AC093673.1 | 1.398390647 | 1.089320977 | 1.795151697 | 0.008505299 |
| AC004923.4 | 1.783020242 | 1.125967574 | 2.823492663 | 0.013669113 |
| AC109454.2 | 1.933764743 | 1.010290557 | 3.701357053 | 0.046495026 |
| AL162431.1 | 1.565849268 | 1.020575056 | 2.402453317 | 0.040051755 |
| FOXD2-AS1 | 1.376499369 | 1.092653531 | 1.734081717 | 0.006687878 |
| AC008622.2 | 2.627413495 | 1.480568385 | 4.66260238 | 0.000963662 |
| AC135050.3 | 1.569899019 | 1.034054566 | 2.383416708 | 0.034246004 |
| AP000240.1 | 1.737509798 | 1.275141352 | 2.367533837 | 0.00046581 |
| AC012368.1 | 1.417304951 | 1.043585711 | 1.924857061 | 0.025539836 |
| AC025265.1 | 1.385857754 | 1.022828352 | 1.877736092 | 0.035238225 |
| AC079209.1 | 1.308687203 | 1.033499345 | 1.657148795 | 0.025514043 |
| AC007998.3 | 1.866264125 | 1.190103619 | 2.926587004 | 0.006564529 |
| LINC01093 | 0.745159353 | 0.61607523 | 0.901290028 | 0.002439432 |
| THUMPD3-AS1 | 1.986457867 | 1.312510946 | 3.006462437 | 0.001169901 |
| AL118511.1 | 1.765051942 | 1.089483849 | 2.859526887 | 0.020992474 |
| AC020915.1 | 1.494985971 | 1.041125424 | 2.146699141 | 0.029384975 |
| AC055822.1 | 1.503410701 | 1.044851478 | 2.163220116 | 0.028070609 |
| LINC01011 | 1.632720609 | 1.034573494 | 2.576691363 | 0.035206721 |
| AC007038.1 | 1.572937794 | 1.028416069 | 2.405770754 | 0.036689259 |
| AL117336.2 | 2.052293349 | 1.430615144 | 2.944123726 | 9.42E-05 |
| AC009041.3 | 1.784905926 | 1.074692187 | 2.964466668 | 0.025204582 |
| AC004801.6 | 2.08187651 | 1.101028849 | 3.936508846 | 0.024065261 |
| AC102953.2 | 1.518624164 | 1.032560046 | 2.233496598 | 0.033774223 |
| AC125494.2 | 1.767150728 | 1.072068047 | 2.91289504 | 0.025557478 |
| AC010973.2 | 1.473155701 | 1.022186412 | 2.123084101 | 0.037741778 |
| AL161729.4 | 1.913323447 | 1.165398197 | 3.141249595 | 0.01031557 |
| PXN-AS1 | 1.499450433 | 1.026516334 | 2.190273574 | 0.036141663 |
| AL137186.1 | 2.186915981 | 1.475968386 | 3.240314328 | 9.59E-05 |
| AC010201.2 | 1.65253397 | 1.023167897 | 2.669032647 | 0.040014315 |
| MIR194-2HG | 1.732418973 | 1.046441102 | 2.868078761 | 0.032642471 |
| AC024257.4 | 3.609711524 | 1.551019172 | 8.400938894 | 0.002897985 |
| DANCR | 1.516281786 | 1.215860232 | 1.890933181 | 0.000220018 |
| AC010173.1 | 2.165339994 | 1.194891308 | 3.923952965 | 0.010866605 |
| AC040970.1 | 1.303879695 | 1.050948751 | 1.617683316 | 0.015882406 |
| SNHG3 | 1.417611862 | 1.122905648 | 1.789663624 | 0.003337217 |
| AC100872.2 | 1.514034137 | 1.100144095 | 2.083635569 | 0.010904484 |
| AC018638.6 | 1.868338148 | 1.038059508 | 3.362704554 | 0.0371114 |
| AC097641.2 | 2.338682895 | 1.260253459 | 4.339950542 | 0.007076179 |
| AL117335.1 | 1.400189438 | 1.054648125 | 1.858942727 | 0.019915361 |
| AC010969.2 | 1.94355339 | 1.282213783 | 2.94599842 | 0.001739914 |
| AC004812.2 | 1.671303714 | 1.048431193 | 2.66422453 | 0.030869455 |
| AL008718.3 | 3.248087953 | 1.604475659 | 6.575403802 | 0.001060815 |
| AL049539.1 | 1.979419871 | 1.123722976 | 3.486716131 | 0.018089435 |
| AP003390.1 | 1.606161282 | 1.03956087 | 2.48158058 | 0.03278087 |
| LINC00205 | 1.553571075 | 1.187843861 | 2.031902646 | 0.001295745 |
| AC010761.1 | 1.531745684 | 1.076542238 | 2.179426647 | 0.01779423 |
| CYTOR | 1.418192602 | 1.163218458 | 1.729056345 | 0.000550053 |
| AL162411.1 | 1.538904377 | 1.168863463 | 2.026093514 | 0.002127236 |
| MYADM-AS1 | 2.235916451 | 1.133644086 | 4.409957619 | 0.020236686 |
| AC007773.1 | 1.285452499 | 1.027297388 | 1.608480804 | 0.028133383 |
| GHRLOS | 2.055769297 | 1.069499168 | 3.951557447 | 0.030657298 |
| AC244034.2 | 2.330865761 | 1.076972295 | 5.044637844 | 0.031697447 |
| AL049840.5 | 1.539775778 | 1.103043931 | 2.149424315 | 0.011205359 |
| AL355802.3 | 1.616108002 | 1.128813965 | 2.31376042 | 0.00874797 |
| LINC01224 | 1.365947482 | 1.013885474 | 1.840259647 | 0.040301669 |
| AL049612.1 | 1.567708685 | 1.099329159 | 2.235645713 | 0.013030629 |
| POLH-AS1 | 2.445366276 | 1.484487798 | 4.028201668 | 0.000445881 |
| AC009005.1 | 1.501510961 | 1.206527121 | 1.868615406 | 0.000270188 |
| TMCC1-AS1 | 2.220409616 | 1.461935633 | 3.372391199 | 0.000183343 |
| LINC02159 | 1.510821235 | 1.046911001 | 2.180300715 | 0.027459659 |
| AC007611.1 | 2.102213664 | 1.040258785 | 4.248272019 | 0.038459515 |
| AC099508.2 | 0.606498452 | 0.437243722 | 0.841270792 | 0.002742033 |
| AC046143.2 | 1.58992411 | 1.169131542 | 2.162167889 | 0.003114604 |
| SNHG7 | 1.376400234 | 1.072261439 | 1.766805684 | 0.012155295 |
| AC004160.1 | 0.290816041 | 0.10719193 | 0.788995677 | 0.015293095 |
| AC073896.4 | 1.412555837 | 1.083911054 | 1.840846611 | 0.010579054 |
| PRRT3-AS1 | 1.383583272 | 1.118242398 | 1.711885253 | 0.002801455 |
| AC019080.5 | 2.173993375 | 1.436538062 | 3.290025736 | 0.000239249 |
| AC005332.5 | 1.637000894 | 1.171959708 | 2.286573429 | 0.003845294 |
| LINC00622 | 1.666304837 | 1.110201618 | 2.500961776 | 0.013718337 |
| AC138356.1 | 0.406833093 | 0.210012158 | 0.788112302 | 0.007681542 |
| LINC01836 | 1.231021973 | 1.016642202 | 1.490608098 | 0.033251488 |
| AC025171.4 | 1.530138645 | 1.066040425 | 2.196280946 | 0.021067031 |
| FARP1-AS1 | 1.500244436 | 1.035401655 | 2.173778027 | 0.032046209 |
| AL355987.4 | 1.486859648 | 1.127771466 | 1.960283338 | 0.004915103 |
| AF230666.1 | 2.86810967 | 1.221530929 | 6.734215963 | 0.015543748 |
| AC008875.1 | 1.761276484 | 1.136279345 | 2.730045974 | 0.011364173 |
| AC124798.1 | 1.362967863 | 1.1026044 | 1.684812247 | 0.004196174 |
| SNHG17 | 1.353093323 | 1.000410479 | 1.830110318 | 0.049689464 |
| AC007292.2 | 4.047814982 | 1.464161703 | 11.19057144 | 0.007042048 |
| SNHG20 | 1.46731625 | 1.015199776 | 2.120781573 | 0.041327176 |
| LINC01929 | 1.513405258 | 1.085360099 | 2.110263201 | 0.014570754 |
| AC012676.1 | 1.854884735 | 1.191295997 | 2.888112935 | 0.006242114 |
| ZNF436-AS1 | 1.827363715 | 1.102798048 | 3.027986992 | 0.019298415 |
| AC126118.1 | 1.853771557 | 1.124526019 | 3.05592661 | 0.015514473 |
| CDKN2B-AS1 | 1.961202608 | 1.250911837 | 3.074809554 | 0.003327755 |
| AL137127.1 | 3.138078927 | 1.461668042 | 6.737192762 | 0.003349566 |
| AC009275.1 | 1.304393953 | 1.090856987 | 1.559731115 | 0.003575477 |
| AC092171.2 | 1.60971149 | 1.235723885 | 2.096885163 | 0.000417193 |
| AC010247.2 | 1.171309038 | 1.001002528 | 1.370590808 | 0.048556711 |
| SREBF2-AS1 | 1.890784444 | 1.321752514 | 2.704792142 | 0.000488375 |
| ZNF433-AS1 | 1.750919412 | 1.028858 | 2.979729745 | 0.038938462 |
| FAM99A | 0.852051111 | 0.746969573 | 0.971915219 | 0.017118386 |
| GSEC | 1.802631105 | 1.263688502 | 2.571423967 | 0.001148773 |
| AP000866.6 | 2.285873328 | 1.069244445 | 4.886830973 | 0.032951512 |
| AC004160.2 | 0.489980775 | 0.256573995 | 0.935718993 | 0.03067623 |
| BACH1-IT2 | 2.365483397 | 1.120391851 | 4.994245267 | 0.023938797 |
| AC010327.6 | 1.943270338 | 1.137607481 | 3.319510173 | 0.015020068 |
| AL080317.2 | 1.898370154 | 1.126570741 | 3.198919614 | 0.016057692 |
| SCAT2 | 1.486336852 | 1.046126997 | 2.11178685 | 0.026993416 |
| AC023090.1 | 1.316993926 | 1.125373821 | 1.541241646 | 0.000598532 |
| AC012073.1 | 1.770270144 | 1.253091013 | 2.500900852 | 0.0011963 |
| MIR4435-2HG | 1.446991137 | 1.115259422 | 1.877395795 | 0.005418565 |
| AL645940.1 | 1.67425134 | 1.038614796 | 2.698900073 | 0.03438845 |
| AC026401.3 | 1.545526514 | 1.209847002 | 1.974342378 | 0.000492704 |
| AC006504.5 | 1.718370413 | 1.125097397 | 2.62448112 | 0.012229312 |
| AC145285.2 | 2.798547296 | 1.494399695 | 5.240811407 | 0.001304544 |
| AL121917.2 | 1.738350581 | 1.075726669 | 2.809136213 | 0.023941656 |
| AL513320.1 | 1.854294797 | 1.189941334 | 2.889561945 | 0.006365673 |
| AC012313.9 | 0.670495786 | 0.485104683 | 0.926737289 | 0.015489905 |
| AC012557.2 | 2.991691849 | 1.17401419 | 7.623604718 | 0.021669577 |
| AL021392.1 | 2.690808801 | 1.599990316 | 4.525309892 | 0.000189972 |
| AC145207.5 | 2.099447245 | 1.437160498 | 3.066935628 | 0.000125334 |
| AC005776.2 | 2.146262597 | 1.182023267 | 3.897083299 | 0.012092424 |
| AL359504.1 | 1.526112191 | 1.038570688 | 2.242522772 | 0.031343117 |
| AC011462.4 | 1.475479981 | 1.013880425 | 2.147236619 | 0.042156285 |
| AC010326.3 | 1.410795639 | 1.020596404 | 1.950177688 | 0.037216566 |
| AC005332.3 | 1.381443284 | 1.002123225 | 1.9043422 | 0.04850537 |
| AC017083.1 | 2.185461555 | 1.10185734 | 4.334719237 | 0.025249278 |
| LINC01353 | 1.880810359 | 1.046847983 | 3.379141635 | 0.034590872 |
| AC025171.5 | 1.565272382 | 1.001997429 | 2.445193529 | 0.048983904 |
| DDX11-AS1 | 3.620778969 | 1.959142906 | 6.691722335 | 4.02E-05 |
| AC084824.4 | 1.510087376 | 1.050740759 | 2.170244053 | 0.025917309 |
| AC125437.1 | 1.966079445 | 1.296250933 | 2.982037108 | 0.001468615 |
| AC026355.2 | 1.878722491 | 1.282575107 | 2.751962188 | 0.001204578 |
| AC253576.2 | 1.620254019 | 1.007904769 | 2.604634055 | 0.046319227 |
| TRIM52-AS1 | 1.547417947 | 1.104049524 | 2.168835954 | 0.01125683 |
| LINC02367 | 2.923045281 | 1.23209766 | 6.934672467 | 0.014954183 |
| MAFA-AS1 | 1.2188396 | 1.044339903 | 1.422496609 | 0.012063237 |
| AC079061.1 | 0.413537628 | 0.221402246 | 0.772410272 | 0.005604089 |
| AC010655.2 | 2.766510369 | 1.618247396 | 4.729548547 | 0.000199798 |
| ITGA9-AS1 | 1.943694344 | 1.220806305 | 3.094633182 | 0.005098278 |
| MIR210HG | 1.510503974 | 1.243266738 | 1.835183221 | 3.30E-05 |
| AC026356.2 | 4.503677476 | 1.728168737 | 11.73676526 | 0.002074247 |
| PPP1R14B-AS1 | 1.265398136 | 1.016959097 | 1.574529838 | 0.034792536 |
| LINC01138 | 1.739898002 | 1.234661379 | 2.451882848 | 0.001554083 |
| AC015961.2 | 1.557681079 | 1.005316624 | 2.413538469 | 0.047289577 |
| AP000553.2 | 1.554631567 | 1.084182947 | 2.229217232 | 0.016417067 |
| AL356481.1 | 2.37879591 | 1.182785615 | 4.784189044 | 0.015063134 |
| LINC01424 | 2.913482755 | 1.232985509 | 6.884413241 | 0.014796183 |
| AL137186.2 | 1.588215101 | 1.198607807 | 2.104464191 | 0.001275071 |
| AC245060.5 | 1.872627471 | 1.065127221 | 3.29231436 | 0.029322221 |
| AATBC | 3.513764567 | 1.304998244 | 9.460964021 | 0.012892642 |
| AC107214.2 | 1.955710299 | 1.085031505 | 3.52506149 | 0.025650631 |
| AL671710.1 | 1.849279962 | 1.022216417 | 3.34551111 | 0.042091856 |
| CDKN2A-DT | 3.275446059 | 1.367475785 | 7.845511419 | 0.007762942 |
| UNC5B-AS1 | 1.862963679 | 1.057196609 | 3.282864926 | 0.031367205 |
| AL121890.5 | 2.011975886 | 1.079923606 | 3.748456783 | 0.027653857 |
| LGALSL-DT | 3.225460897 | 1.535064193 | 6.777304851 | 0.001993139 |
| AC069234.4 | 1.805047176 | 1.032912601 | 3.154376571 | 0.038110382 |
| AC004477.2 | 1.722868015 | 1.015581683 | 2.922733097 | 0.043664153 |
| AP001469.3 | 1.734900014 | 1.16639788 | 2.580489995 | 0.006532099 |
| AC006538.1 | 1.645136496 | 1.130366035 | 2.394334228 | 0.009323604 |
| AC011446.1 | 2.743733297 | 1.109429226 | 6.785536408 | 0.028907512 |
| ZFAS1 | 1.285850296 | 1.018874566 | 1.622781683 | 0.034222297 |
| AL121906.2 | 1.521418329 | 1.078039296 | 2.147151538 | 0.016964258 |
| AP001972.1 | 2.459285647 | 1.152829232 | 5.246298173 | 0.019919028 |
| MELTF-AS1 | 1.363150304 | 1.082060963 | 1.717258837 | 0.008555307 |
| SNHG12 | 1.398470902 | 1.028296606 | 1.901903451 | 0.032530368 |
| LINC00685 | 1.535095415 | 1.134128254 | 2.077823141 | 0.00552269 |
| AP003392.3 | 1.761232641 | 1.156704669 | 2.681704758 | 0.008325079 |
| AC107214.1 | 1.984366321 | 1.117928011 | 3.522328502 | 0.019246196 |
| LINC02613 | 2.636425055 | 1.167313561 | 5.954472989 | 0.019693493 |
| AL358072.1 | 2.054001939 | 1.077604631 | 3.915094502 | 0.028738052 |
| AC113410.3 | 1.371110477 | 1.089453633 | 1.725584167 | 0.007140119 |
| AC092667.1 | 1.954868308 | 1.046932355 | 3.650197728 | 0.035385725 |
| ZNF529-AS1 | 1.543897444 | 1.083508607 | 2.19990806 | 0.016221498 |
| ELF3-AS1 | 1.306294103 | 1.0245118 | 1.665577969 | 0.031138192 |
| AP000569.1 | 1.907065271 | 1.151364939 | 3.158770798 | 0.012161785 |
| LINC02313 | 1.32133544 | 1.059105253 | 1.648492763 | 0.013559106 |
| AC020915.2 | 1.996451472 | 1.320977687 | 3.017324607 | 0.001034286 |
| LINC02804 | 1.732322646 | 1.25474166 | 2.391680971 | 0.00084091 |
| AP000238.1 | 2.07633263 | 1.24045892 | 3.475453415 | 0.005438575 |
| LINC01063 | 1.857292356 | 1.346907653 | 2.561077509 | 0.000158985 |
| SOX9-AS1 | 1.493448388 | 1.159918896 | 1.922882794 | 0.001868285 |
| AP003392.4 | 2.311749587 | 1.157308295 | 4.617772271 | 0.017605515 |
| LINC00862 | 1.643991492 | 1.128673962 | 2.394587026 | 0.009575778 |
| Z97989.1 | 2.411835759 | 1.18429788 | 4.911730256 | 0.015262395 |
| MAPKAPK5-AS1 | 1.684825902 | 1.198487602 | 2.368517051 | 0.002683349 |
| AC026803.1 | 1.637685817 | 1.143630246 | 2.345176549 | 0.00709152 |
| U91328.3 | 2.039570965 | 1.196564843 | 3.47649335 | 0.008805657 |
| SNHG1 | 1.417245178 | 1.082047937 | 1.856279952 | 0.011319297 |
| AL355574.1 | 1.58656743 | 1.196268022 | 2.104207555 | 0.001355906 |
| AC006504.7 | 1.503800004 | 1.108492766 | 2.040080478 | 0.008744767 |
| ZNNT1 | 1.365296702 | 1.029694273 | 1.81028013 | 0.030521042 |
| AC048344.4 | 3.735274719 | 1.823214821 | 7.652569001 | 0.000316701 |
| U62317.1 | 1.418144015 | 1.076787729 | 1.867714864 | 0.01289906 |
| AL353708.3 | 1.691464254 | 1.063968174 | 2.689038445 | 0.026275804 |
| AC092384.2 | 0.439712391 | 0.206260614 | 0.937391698 | 0.033389732 |
| SNHG6 | 1.267067274 | 1.038651775 | 1.545714853 | 0.019601713 |
| AP001505.1 | 1.330689277 | 1.071583794 | 1.652445624 | 0.009718342 |
| AL451069.3 | 1.428414977 | 1.109506027 | 1.838988971 | 0.005673199 |
| AL161668.4 | 0.780068269 | 0.644884442 | 0.943589989 | 0.010529194 |
| AC034229.4 | 1.877056439 | 1.285202761 | 2.741466938 | 0.001120894 |
| TFAP2A-AS1 | 1.462972305 | 1.077183098 | 1.986930513 | 0.014851006 |
| AC026356.1 | 2.42944124 | 1.51713023 | 3.890361303 | 0.000219836 |
| AL121917.1 | 2.235906577 | 1.303575785 | 3.835049927 | 0.003466437 |
| AC025176.1 | 1.746001926 | 1.183706487 | 2.575404257 | 0.004947862 |
| AC132192.2 | 1.623948379 | 1.148922312 | 2.295375684 | 0.006027684 |
| UPK1A-AS1 | 1.434475276 | 1.136950597 | 1.809858162 | 0.002348697 |
| GAS5 | 1.285347825 | 1.028821973 | 1.605835678 | 0.027095963 |
| AC119403.1 | 2.065794328 | 1.163478182 | 3.667886748 | 0.013253389 |
| AC116025.2 | 1.790440832 | 1.195938969 | 2.68046987 | 0.004668747 |
| LNCSRLR | 2.085202109 | 1.367792381 | 3.178894615 | 0.000636072 |
| MYLK-AS1 | 1.485040149 | 1.075290204 | 2.050929355 | 0.016366133 |
| AL080317.1 | 1.962027734 | 1.205680552 | 3.192846416 | 0.006670985 |
| ELFN1-AS1 | 1.240612805 | 1.083678781 | 1.420273386 | 0.00178066 |
| BACE1-AS | 1.779504582 | 1.260046005 | 2.513111859 | 0.001066313 |
| AC010501.2 | 0.49782185 | 0.256947172 | 0.964504075 | 0.038727758 |
| AC073573.1 | 0.355101298 | 0.191737337 | 0.657654545 | 0.000992081 |
| LINC01532 | 2.398169283 | 1.384246839 | 4.154761817 | 0.001810777 |
| LUCAT1 | 1.493786783 | 1.188240779 | 1.877901343 | 0.000587845 |
| AC092115.3 | 1.39402785 | 1.011619342 | 1.920992971 | 0.042297766 |
| LINC02806 | 1.222717953 | 1.023240145 | 1.461083403 | 0.026912206 |
| AC090772.3 | 1.907480359 | 1.379497913 | 2.637540285 | 9.39E-05 |
| LINC01269 | 1.401762588 | 1.116513989 | 1.759886908 | 0.003621467 |
| LINC00942 | 1.278336359 | 1.122444781 | 1.455879054 | 0.000214938 |
| LINC02561 | 1.430233809 | 1.058489753 | 1.932535239 | 0.019800608 |
| AC145207.8 | 1.495650541 | 1.079061531 | 2.073070421 | 0.015658413 |
| AC008750.2 | 1.652781074 | 1.147672514 | 2.380195785 | 0.00693114 |
| AC124067.4 | 1.438833568 | 1.121011259 | 1.846762929 | 0.004277338 |
| AL355482.1 | 0.624897443 | 0.4078834 | 0.957373637 | 0.030764969 |
| AL359313.1 | 1.851840637 | 1.280469101 | 2.678169856 | 0.001063047 |
| LINC01344 | 0.408219913 | 0.180989361 | 0.920736424 | 0.030853294 |
| AC010531.5 | 0.614915429 | 0.386836442 | 0.977469918 | 0.039750884 |
